# Understanding and Controlling Reactivity Patterns of Pd_1_@C_3_N_4_-Catalyzed Suzuki–Miyaura Couplings

**DOI:** 10.1021/acscatal.4c03531

**Published:** 2024-08-07

**Authors:** Marc Eduard Usteri, Georgios Giannakakis, Aram Bugaev, Javier Pérez-Ramírez, Sharon Mitchell

**Affiliations:** † Department of Chemistry and Applied Biosciences, Institute of Chemical and Bioengineering, ETH Zurich, Vladimir-Prelog-Weg 1, Zurich 8093, Switzerland; ‡ Paul Scherrer Institute, Forschungsstrasse 111, Villigen 5232, Switzerland

**Keywords:** single-atom catalysis, palladium, carbon nitride, Suzuki−Miyaura coupling, mechanism, in situ XAS

## Abstract

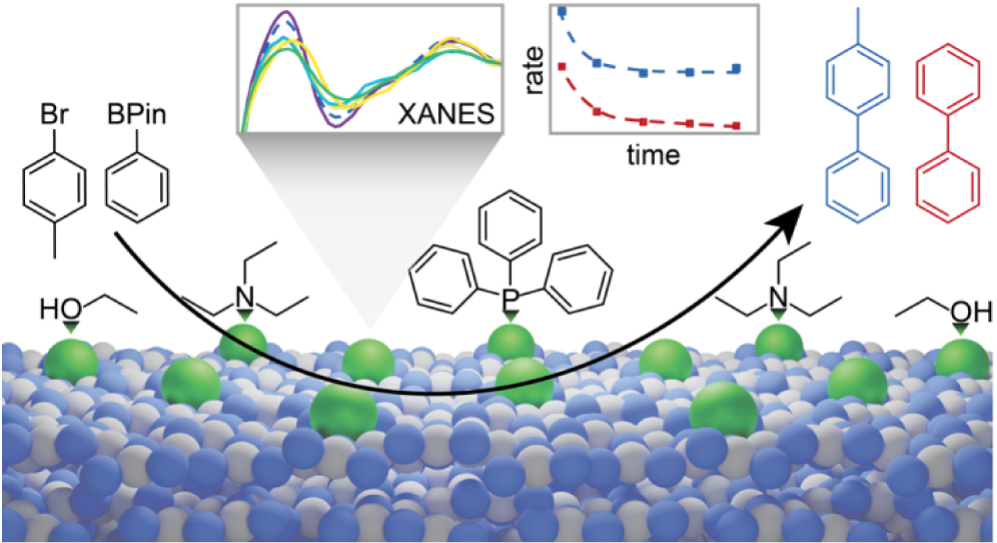

Using heterogeneous single-atom catalysts (SACs) in the Suzuki–Miyaura coupling (SMC) has promising economic and environmental benefits over traditionally applied palladium complexes. However, limited mechanistic understanding hinders progress in their design and implementation. Our study provides critical insights into the working principles of Pd_1_@C_3_N_4_, a promising SAC for the SMC. We demonstrate that the base, ligand, and solvent play pivotal roles in facilitating interface formation with reaction media, activating Pd centers, and modulating competing reaction pathways. Controlling the previously overlooked interplay between base strength, reagent solubility, and surface wetting is essential for mitigating mass transfer limitations in the triphasic reaction system and promoting catalyst reusability. Optimum conditions for Pd_1_@C_3_N_4_ require polar solvents, intermediate base strength, and increased water content. Our investigations reveal that high selectivity requires minimizing competitive coordination of bases and phosphine ligands to the Pd centers, to avoid homocoupling and alternative reductive elimination mechanisms giving rise to phosphonium side-products. Furthermore, in situ XAS investigations probing electronic structures and coordination environments of Pd sites further rationalize the base and ligand coordination, confirming and expanding upon previous computational hypotheses for Pd_1_@C_3_N_4_. This understanding allows for designing a more selective ligand-free reaction pathway using the solvent and base to modulate the chemical environment of the active sites. We highlight the importance of environment-compatible surface tension, the creation of coordinatively available active sites, and the stabilization of partially reduced Pd centers, emphasizing the importance of mechanistic studies to advance the design of SACs in organic liquid phase reactions.

## 1 Introduction

**Figure 1:**
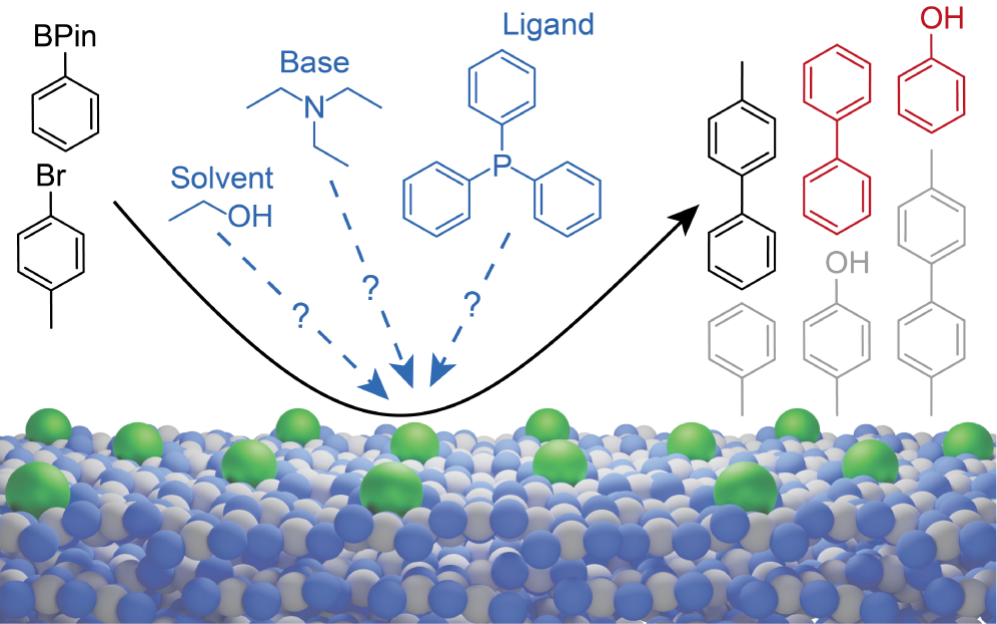
Scheme of the Pd_1_@C_3_N_4_ catalyzed SMC (black product). Possible side products (red products formed from the boronic acid ester, gray products formed from the aryl halide), as well as unknown interactions with the solvent, base, and ligand (blue dashed arrows) are highlighted.

The discovery and development of palladium-catalyzed cross-coupling chemistry is one of the greatest chemical achievements of the last 50 years.^1−3^ Widely used in the fine and specialty chemical sectors, it is the only reaction family developed over that period commonly used in medicinal chemistry.^4,5^ Nevertheless, palladium metal complexes are expensive and have a high ecological footprint if the metal is not recovered, as quantified by a recent life cycle analysis study,^6^ necessitating efficient use and recovery. Heterogeneous single-atom catalysts (SACs), which may help address these challenges, have shown promise for the Suzuki–Miyaura coupling (SMC).^7−12^ Due to the early stage of the field, most studies focus on developing and characterizing new materials, treating them as drop-in solutions tested under model conditions, and assuming identical reaction paths as homogeneous analogs. The interactions with various reaction components (substrates, solvents, bases, and ligands) and related structural modifications and mechanistic implications remain mostly unexplored. Importantly, selectivity aspects beyond the limiting reagent, such as boronic acid homocoupling and substrate degradation (Figure 1), are missing despite their importance to process design.^13^ The selection of the reaction medium is also tied to other synthesis and separation steps, making tolerance toward various conditions a desirable catalyst property.

For the SMC with Pd complexes, selecting bases, solvents, and ligands is key to facilitating the reaction and maximizing yields, as each component affects the overall cycle differently. Generally, higher basicity helps generate hydroxide ions responsible for either coordination to Pd after the oxidative addition or boronic acid activation.^14^ Water is a necessary cosolvent in most conditions, while carbonate and phosphate bases are often preferred. However, this simplified view often neglects other structural or electronic effects of the base.^14,15^ Solvent polarity should facilitate the formation and solubility of the most active Pd species.^16^ Consequently, aromatic (toluene, xylenes) and ethereal solvents (1,4-dioxane, dimethoxymethane) complement uncharged Pd species, while polar solvents are preferred for anionic precursors and intermediates.^16^ Electron-donating, bulky ligands generally facilitate the formation of highly reactive 12 or 14 valence electron intermediates required for oxidative addition,^17^ and are essential to reduce Pd(II) precursors to catalytically active Pd(0) species.

It is unclear whether these trends hold when moving toward SACs. The importance of mechanistic investigations was exemplified in the Heck coupling,^18^ where the careful selection of the base could shift the rate-limiting step away from the oxidative addition and determine the chemoselectivity between coupling products. Concerning solvents, heterogeneous catalysts are uncharged and do not require solubilization. Notably, many reported SAC systems show preferred or exclusive activity in alcoholic solvents.^8−11^ Unlike metal complex catalysts, solid catalysts form triple-phase systems with common solvents that may suffer from mass-transfer limitations. Carriers also influence the electronic state and coordination environment of the Pd atoms in addition to or instead of exchangeable ligands, which are typically phosphines. Palladium single atoms anchored on carbon nitride (Pd_1_@C_3_N_4_) were the first SAC reported for the SMC, delivering efficient and stable performance when used with triphenylphosphine ligands.^7^ Ligand-free operation was also reported in more recent studies on SACs, primarily for oxide-based systems such as Pd_1_@CeO_2_,^8^ Pd_1_@ZnO-ZrO_2_,^9^ and Pd_1_@FeO_*x*_.^10^ Nonetheless, the working principles of these systems, including the role of the carrier and ligands, remain unclear and are mostly limited to theoretical insights from density functional theory (DFT) simulations.^19^ This gap underscores the urgent need for mechanistic studies to advance the design and implementation of SACs, essential for developing effective and sustainable catalytic technologies.

To address this, we investigate factors that influence reactivity patterns of Pd_1_@C_3_N_4_ in the SMC. By mapping the performance across various solvents and bases, we identify a trade-off between base strength, solubility, and surface wetting, establishing optimal conditions to reduce mass-transfer limitations and mitigate fouling-related deactivation pathways. The choice of base is shown to be essential for enhancing selectivity, as coordinating bases compete with the triphenylphosphine ligand (TPP) to bond to palladium centers, promoting alternative reductive elimination paths. In situ X-ray absorption spectroscopy (XAS) corroborated our hypothesis for base coordination on palladium sites, revealing that the metal reduction differs from that observed for metal complex catalysts and can be induced by TPP or ethanol. Building on this knowledge, we identify conditions for phosphine-free couplings, revealing a dual role of TPP. Furthermore, the kinetic analysis shows that transmetalation, rather than oxidative addition, limits the catalytic cycle. These insights reveal new opportunities for catalyst design.

## 2 Methods

### 2.1 Chemicals

All commercial reagents (Table S1) were used as received without further purification.

### 2.2 Catalyst Preparation

Pd_1_@C_3_N_4_ was synthesized following a previously reported protocol.^7^ A high surface area graphitic carbon nitride (C_3_N_4_) was prepared via the polymerization of dicyandiamide and exfoliation of the resulting material. Powdered dicyandiamide was placed in a ceramic crucible at 550 °C for 4 h (2.3 °C min^–1^ temperature ramp) in static air. The resulting material was crushed and treated at 500 °C for 10 h (5 °C min^–1^ ramp) in static air. Palladium was subsequently stabilized on C_3_N_4_ by wet deposition followed by thermal activation. Exfoliated carbon nitride (2 g) was sonicated in deionized water (DIW, 20 mL) for 30 min followed by the addition of an aqueous solution of (NH_3_)_4_Pd(NO_3_)_2_ (284.4 mg, 10 wt % complex, 3.66 wt % Pd) diluted in DIW (3 mL). The resulting suspension was stirred for 16 h and subjected to a cyclic microwave treatment in a CEM Discover SP consisting of 15 s irradiation at 100 W, followed by 3 min cooling with 20 repetitions. Then, the powder was filtered off, washed with DIW, and dried overnight at 80 °C. Finally, the catalyst was activated at 300 °C (5 °C min^–1^ temperature ramp) in flowing nitrogen for 5 h, resulting in Pd_1_@C_3_N_4_. The synthesis of other carriers and catalysts is detailed in the Supporting Information.

### 2.3 Catalyst Characterization

A Horiba Ultra 2 instrument equipped with a photomultiplier tube detector was used for inductively coupled plasma-optical emission spectrometry (ICP-OES). To determine the metal loading, the catalysts were mixed with nitric acid (65%) and digested at 220 °C for 30 min using an Anton Paar Multiwave 7000. Results can be found in Table S2. Pd leaching was determined by separating the solid catalyst from the reaction mixture by filtration, evaporating the solvents at 80 °C, adding 2 mL of hydrogen peroxide (>30%) and 0.5 mL of nitric acid to the residue, and digesting the suspension at 200 °C for 30 min. High-angle annular dark field scanning transmission electron microscopy (HAADF-STEM) and energy dispersive X-ray spectroscopy (EDX) measurements were performed on a Talos F200X instrument operated at 200 kV and equipped with an FEI SuperX detector. High magnification micrographs were instead acquired on a Jeol GrandARM operated at 300 kV. Contact angles between catalysts and reaction mixtures were attempted to be measured on an Automatic Microscopic Contact Angle Meter MCA-3 by Kyowa Interface Science CO. Ltd. Before the measurement, the catalysts were pressed into pellets.

### 2.4 XAS Measurements

Pd *K*-edge (*E*_0_ = 24.3503 keV) X-ray absorption near edge structure (XANES) measurements were conducted at the SuperXAS beamline of the Swiss Light Source^20^ at the Paul Scherrer Institute (PSI), Villigen, Switzerland, and at the Swiss-Norwegian beamlines (SNBL, BM31) of the European Synchrotron Radiation Facility (ESRF), Grenoble, France. At SuperXAS, a Si(111) channel-cut Quick-EXAFS monochromator oscillating at 1 Hz was used to select the incident photon beam, which was provided by a 2.9 T superbend magnet.^20^ At SNBL, a double-crystal liquid nitrogen-cooled Si(111) monochromator was used to collimate the X-ray beam.^21^ Ex situ reference samples were prepared by making pellets from catalyst powder and drying them under N_2_ flow. For in situ measurements, the reaction vials were moved from the heating stage onto the motorized stage after a 2 h reaction time. Regular glass vials were used to minimize the absorbance of the Pd *K*-edge radiation by the vial walls. To optimize the optical pathway through the sample, the vials were aligned along the direction of a focused X-ray beam (slit size ca. 0.3–0.6 mm) instead of perpendicular to it to maximize the optical pathway through the sample. Multiple spectra were measured for 15–20 min and subsequently averaged to maximize the signal-to-noise ratio. The signals were collected in transmission mode through ionization chambers filled with Ar/N_2_ (ca. 10% absorption). The ProQEXAFS software package was used to process the raw data,^22^ which was subsequently analyzed with the Demeter package.^23^ The fit quality is exemplified in Figure S1. The statistical validity of the models was ascertained using the R-factor.

### 2.5 Catalyst Evaluation

In a typical reaction, 1 mol % Pd (e.g., 21.3 mg Pd_1_@C_3_N_4_) was added to a 2 mL vial equipped with a stir bar. A 1 M base solution (0.3 mL) was prepared in deionized water as well as a solution containing bromotoluene (0.1 mmol, 1 equiv), mesitylene (0.1 mmol, 1 equiv), triphenylphosphine (0.01 mmol, 0.1 equiv), and phenylboronic acid pinacol ester (0.15 mmol, 1.5 equiv) in 1 mL of organic solvent. When using triethylamine, the aqueous base solution had to be cooled to 4 °C to prevent phase splitting. The organic solution was added to the catalyst followed by the base solution. Afterward, the vial was placed in an aluminum block and heated to 80 °C under stirring. After 16 h, the stirring was stopped, and the vial was placed in ice to quench the reaction. After cooling down, the solids were removed by syringe microfiltration, and the organic phase was pipetted off and diluted with acetonitrile for analysis by gas chromatography without further purification. For the stability tests, all amounts were doubled, and the catalyst was removed by centrifugation. In some cases, the catalyst was washed by stirring it in 4 mL of DIW for 40 min.

### 2.6 Product Characterization

Product quantification was carried out on a HP 6890 series gas chromatography (GC) system equipped with a DB-5HT column and a flame ionization detector (FID). Mesitylene was used as an internal standard (IS) and conversion *X* and yields *Y*_3_ and *Y*_4_ were calculated according to eqs 1 and 2, where *c*_*n,t*_ designates the concentration of analyte *n* at time *t*. The concentration ratio between analyte and internal standard was obtained from the intensity ratio following a calibration using eq 3, where *m* is the slope of the calibration and relates to the ratio of response factors. Qualitative product analysis was carried out on an Agilent 1260 Infinity high-performance liquid chromatography (HPLC) system with a UV detector (210, 220, and 250 nm) and an Eclipse Plus C18 column. GC coupled to mass spectrometry (MS) was carried out on an Agilent GC-5975 MSD 1 with an Agilent 19091S–433 column. Nuclear magnetic resonance spectroscopy was performed on a Bruker 300 MHz spectrometer at room temperature. ^31^P (with comp pulse decoupling) and ^11^B NMR spectra were recorded by mixing 0.4 mL reaction mixture (filtered organic phase) with 0.15 mL of acetonitrile-d3. For the aqueous phase, D_2_O was used instead.
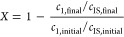
1
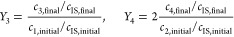
2
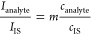
3

## 3 Results and Discussion

### 3.1 Catalyst–Solvent–Base Interplay Controls Activity

**Figure 2:**
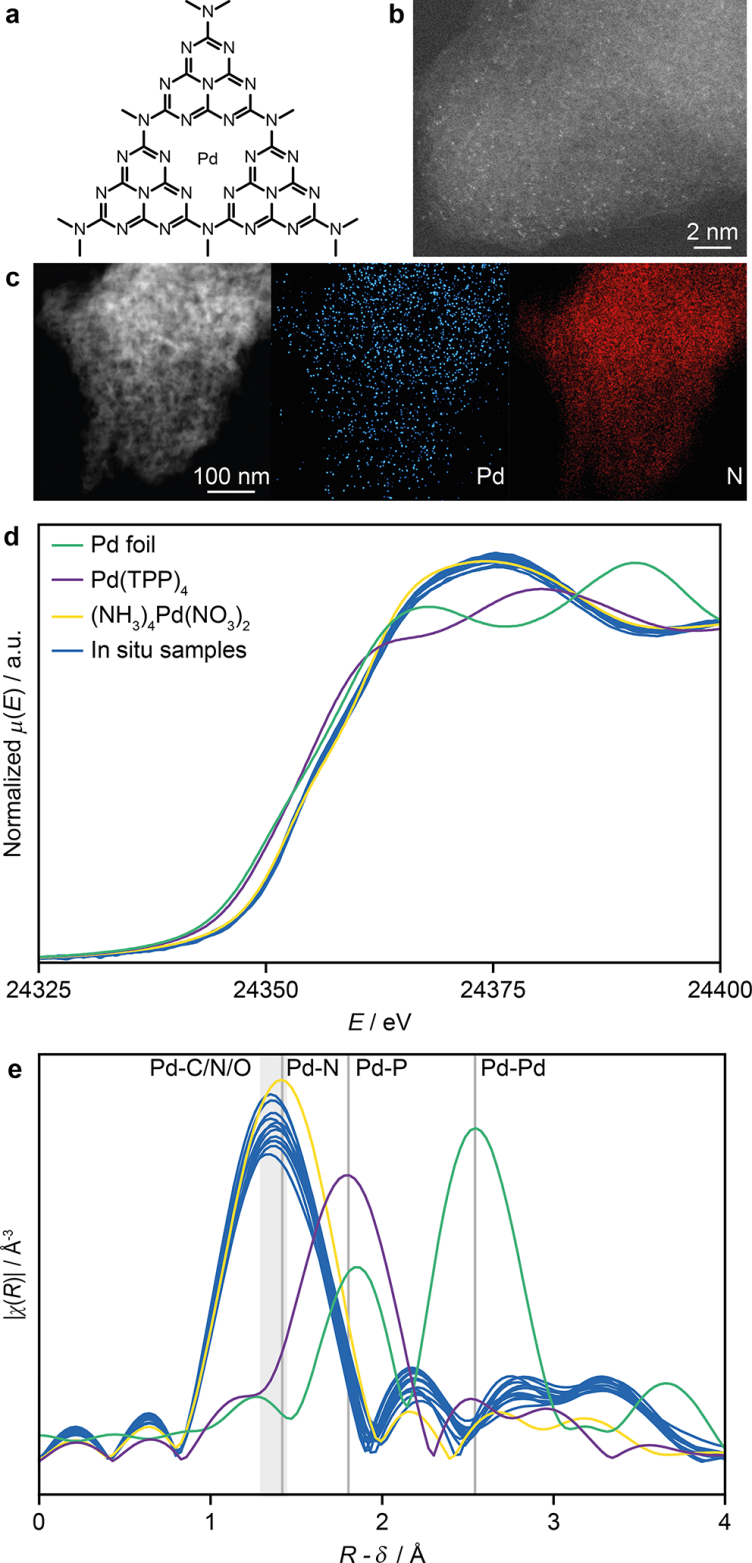
(a) Scheme of the Pd_1_@C_3_N_4_ structural motif. (b) Aberration-corrected HAADF STEM image and (c) EDX elemental mapping. (d) Comparison of all measured in situ Pd K-edge XANES spectra with ex situ reference systems and (e) the corresponding EXAFS spectra. The gray lines and area indicate the bond distances of Pd to its neighbors in reference compounds.

To investigate the mechanism of the surface-catalyzed SMC coupling, we chose to focus on a Pd_1_@C_3_N_4_ catalyst previously reported for the SMC (Figure 2a).^7^ The semicrystalline structure of the exfoliated carbon nitride (C_3_N_4_) host, offers more well-defined metal coordination sites, predominantly comprised of pyridinic nitrogen,^24^ compared to SACs based on other commonly reported host materials, such as heteroatom-doped carbons.^25−28^ Following its successful synthesis via the polymerization of a dicyandiamide precursor (see Section 2), a wet impregnation procedure enabled the introduction of palladium single atoms with 0.52 wt % Pd content, as confirmed by ICP-OES. STEM confirmed the presence of spatially isolated metal atoms (Figure 2b), while EDX elemental mapping demonstrates the uniform distribution of Pd and N across the catalyst (Figures 2c and S2). Finally, in situ XANES and EXAFS analyses of Pd_1_@C_3_N_4_ in solutions containing combinations of all reagents investigated (Figure 2d,e) showed a distinctly different signal from reference Pd(0) samples (metallic foil and Pd(TPP)_4_). Instead, high similarity with the Pd(II) reference is observed in all cases (Table S3), suggesting the prevalence of a formal Pd(II) oxidation state, although the bond distance of the first shell is shifted to lower *R*-values (Table S4). Differences among in situ measurements are discussed in detail in Section 3.3.

**Figure 3:**
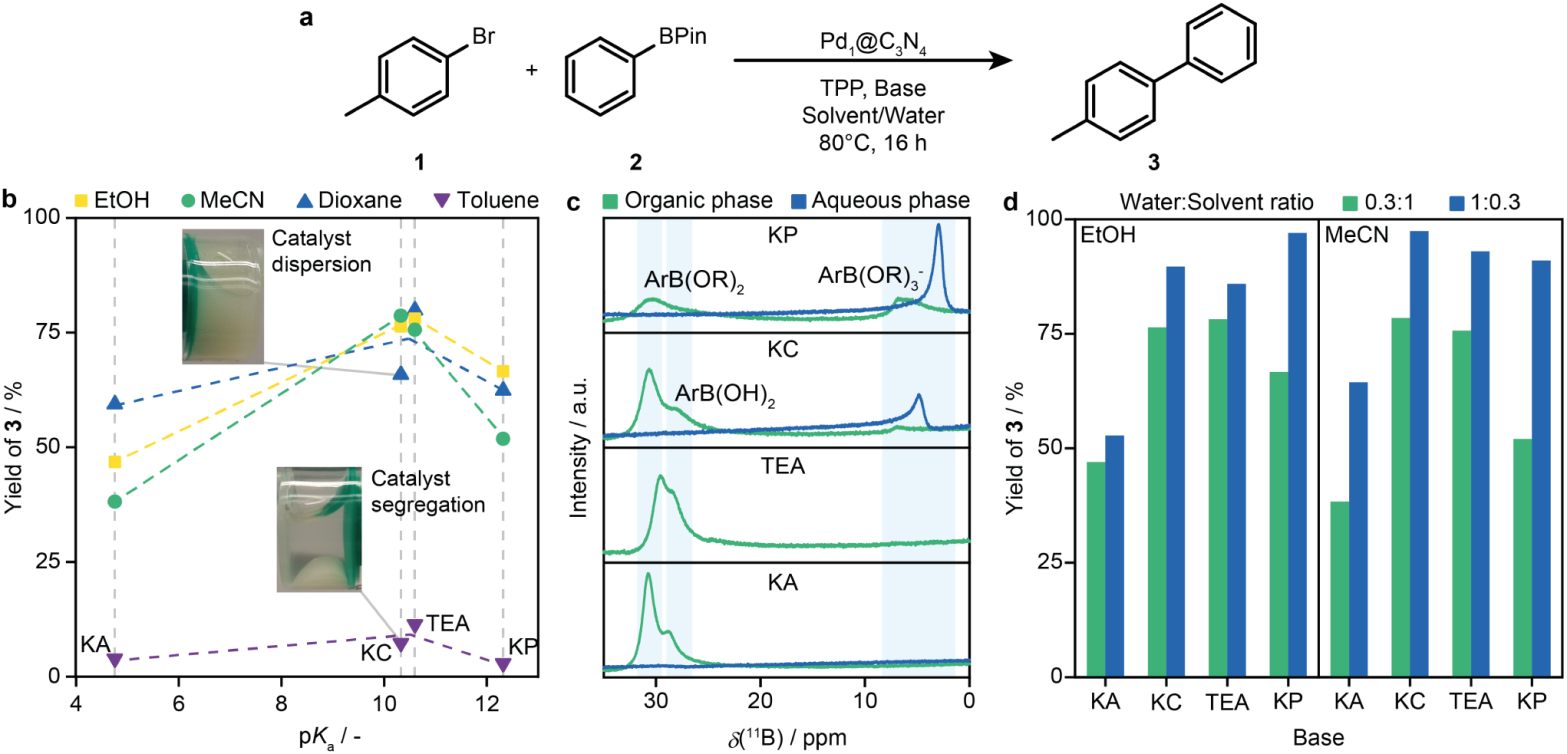
(a) Scheme of the SMC of bromotoluene **1** and phenylboronic acid pinacol ester **2** over Pd_1_@C_3_N_4_. (b) GC yield of 4-methylbiphenyl **3** as a function of base p*K*_a_ in water for each solvent. Insets show the catalyst dispersion in toluene/K_2_CO_3_ (bottom) and dioxane/K_2_CO_3_ (top) while the dashed gray lines indicate the corresponding base. Reaction conditions: 0.1 mmol **1**, 1.5 equiv **2**,1 mol % Pd, 10 mol % TPP, 3 equiv base, 1:0.3 organic to aqueous, 80 °C, 16 h. (c) ^11^B-NMR spectra of the reaction mixtures after 30 min of stirring without catalyst. Mixtures with TEA are monophasic. (d) Effect of the inversion of the solvent-to-water ratio on the yield of **3** for the different bases. All other conditions remain unchanged.

The study initially focused on assessing the effects of base and solvent in the coupling of bromotoluene **1** and phenylboronic acid pinacol ester **2** to 4-methylbiphenyl **3** (Figure 3a) over Pd_1_@C_3_N_4_. This reaction was chosen to prevent strong electronic or solubility effects while still allowing for the discrimination of homocoupling products (biphenyl and 4,4-dimethylbiphenyl). Four commonly used bases and solvents were selected to cover a range of properties. The bases include potassium acetate (KA), potassium carbonate (KC), potassium phosphate (KP), and triethyl amine (TEA), with p*K*_a_ values of the conjugate acid ranging from 4.75 to 12.5. Regarding solubility, TEA is easily soluble in organic solvents followed by KA, KC, and KP, whereas in water KA is the most soluble followed by KC, KP, and TEA. The selected solvents were ethanol (EtOH) as a representative polar protic solvent, acetonitrile and dioxane as polar aprotic solvents, and toluene as an apolar solvent. Their relative polarities compared to water range from 0.099 to 0.654, respectively.^29^ As the hydroxy ions formed from the acid–base equilibrium are believed to be key for the reaction,^30−34^ the base was completely dissolved in water prior to addition to the reaction mixture, and the p*K*_a_ of the conjugate acid in water was chosen as a descriptor. Among all possible combinations, the highest yields were achieved with either KC or TEA in combination with any solvent except toluene (Figure 3b and Table S5).

More generally, reactions in toluene yielded only limited amounts of product **3**, contrasting with previous applications of carbon nitride-based catalysts in hydrogenation and oxidation reactions carried out in toluene.^35−39^ Herein, the presence of an aqueous phase required for the SMC competes with toluene to wet the catalyst surface, which in turn impacts the transport of reagents to and from the active sites. Quantifying surface wetting through contact angles proved challenging due to the porosity of the catalyst and solvent volatility (Note S1). Instead, visual inspection of the reaction mixtures offers qualitative insights (Figure 3c). When the catalyst is agitated in a toluene-based reaction mixture, it remains coated in water and quickly forms a single droplet when the shaking ceases. In contrast, for the other solvents, the catalyst disperses similarly in both phases. Complementary observations were previously reported in solvent-free SMCs with nanoparticle-based catalysts,^40^ where hydrophilic carriers were preferentially wetted by the aqueous phase, thereby limiting contact with the reagents. Thus, using a more hydrophobic carrier seemed like a promising strategy to achieve reactivity. Although Pd single-atoms supported by more hydrophobic nitrogen-containing carbons showed no activity for this reaction, comparable performance was achieved in toluene when using a hydrophobic sulfur-doped carbon system (Table S6), whose synthesis was previously reported.^28^ Importantly, the catalyst could penetrate both phases similar to carbon nitride with the more polar solvents (Figure S3).

By comparing the yields with the p*K*_a_ descriptor identified in the introduction, a trade-off became apparent. As expected, an initial increase in basicity led to higher yields, independent of the solvent used (Figure 3b). However, this trend does not extend to KP, despite having shown the best performance in other SMC systems.^41−45^ The decreased activity is likely due to increased mass transport limitations, possibly from an earlier transition to a mass transport controlled regime or more severe diffusion limitations compared to TEA and KC systems. Additionally, the relatively low solubility of phosphate in all solvents, coupled with the high ionic concentration, hinders intermixing and diffusion across the aqueous–organic interface. Since reagent **2** progressively moves toward the aqueous phase with stronger ionic bases, as observed in ^11^B-NMR measurements (Figure 3c), it differs from the other bases, where it mostly remains in the organic phase as both the starting boronic acid ester and its hydrolyzed version. To alleviate these effects, the solvent ratio was inverted from 0.3:1 aqueous to organic to 1:0.3. This resulted in an emulsion of organic droplets in a mixed aqueous–organic phase (Figure S4). The droplets had a smaller volume than the initially added organic solvent, meaning that parts of the organic and aqueous phases intermixed while simultaneously increasing the interfacial area between the two phases. Furthermore, they could only be aggregated by centrifugation, although the resulting droplet would quickly disintegrate upon slight movement of the vial, showing a preference for the high interfacial area system. Using these modified conditions, the conversion of **1** to **3** increased for all bases, with the most pronounced improvement observed for KP (Figure 3d and Table S7), highlighting the importance of the base solubility and enlargement of the aqueous–organic interface. Surprisingly, the previously monophasic TEA mixture increased in activity, despite also forming a biphasic emulsion through the inversion of the solvent ratio, potentially hinting at an additional promotional effect due to increased water content. Further ^11^B-NMR measurements (Figure S4) show that parts of **2** are now solubilized as a borate in the aqueous phase using TEA, which at least confirms the higher amounts of essential hydroxide ions. Moving toward predominantly aqueous reaction media was identified to be beneficial for process safety and environmental impacts,^46^ an approach that this catalytic system would also benefit from. Nonetheless, complete removal of the organic solvent is challenging with heterogeneous catalysts, as ligands and most reagents are solid under reaction conditions and insoluble in water. The resulting suspension would be unlikely to enable sufficient contact between the catalyst and the reagents to achieve practically relevant yields.

### 3.2 Base-Induced Selectivity Loss

**Figure 4:**
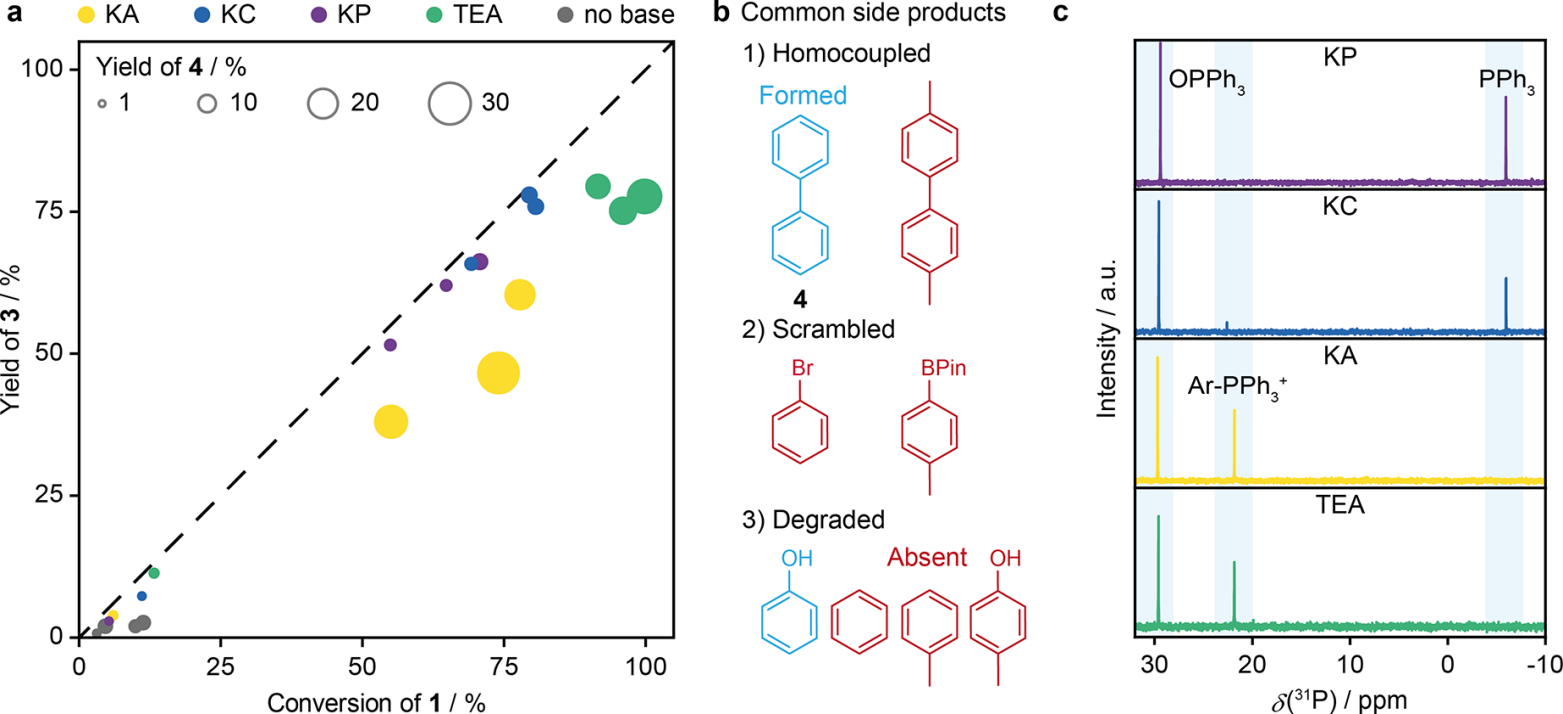
(a) Conversion-yield map of the tested combinations with Pd_1_@C_3_N_4_ using the same conditions as in Figure 3a. The size of the circles represents the GC yield of the main side product biphenyl **4** based on the initial amount of **2**. (b) List of side products encountered in SMC, indicating those that could be identified (blue) and those that were absent (red). (c) ^31^P NMR spectra of crude reaction mixtures. Highlighted areas correspond to regions where triaryl phosphines, tetraaryl phosphonium, and triaryl phosphine oxides are commonly visible.

Based on the previous considerations, the high miscibility of TEA in organic solvents could be expected to give it advantages over ionic bases under conditions with limited water amounts. However, the observed yields are only slightly higher. Comparison of the conversion-yield trends identifies a key issue (Figure 4a). When TEA or KA are used, the yields deviate significantly from the ideal line, indicating that significant portions of **1** are transformed into undesired products. More surprisingly, the deviation in yield of the heterocoupling product seems to be correlated with the yield of the homocoupling of **2** to form biphenyl **4** (Figure S5), suggesting that under these conditions the formation of the homocoupling product **4** is not completely independent of the cross-coupling to form **3**, or that both effects have a common cause. This is further supported by the formation of only traces of **4** in the absence of **1** when using TEA (Figures S6 and S7). The oxidative homocoupling of boronic acids was previously reported using Pd colloids.^47^ However, under the conditions where TEA was employed neither showed significantly more leaching (Table S5) nor nanoparticle formation (Figure S8). Consequently, higher homocoupling yields cannot be attributed to an increased nanoparticle formation rate with TEA but must have a different origin.

Previous studies have linked homocoupling to two main factors, the use of Pd(II) precursors and the presence of oxygen in the solvent.^13,48^ The former facilitates two subsequent transmetalation steps followed by reductive elimination, while the latter can reoxidize the resulting Pd(0) to Pd(II). Since the solvents were not degassed and Pd species present in Pd_1_@C_3_N_4_ exhibit an oxidation state close to a formal Pd(II) based on the XAS analysis of the catalyst, a certain level of homocoupling can be expected, likely corresponding to the levels observed when using KC and KP. Nonetheless, the differences observed when changing bases (i.e., KA, TEA) cannot be explained solely by this reasoning. Alternatively, Lei and Zhang suggested that homocoupling might result from halide/boron scrambling after the transmetalation step.^49^ However, only a few side products could be identified, indicating that this mechanism might not be prevalent (Figures 4b, S6, and S7). Notably, no significant formation of homocoupling or scrambling products beyond trace amounts was observed for **1**. Furthermore, degradation products from **1**, for example, resulting from dehalogenation to toluene, were not formed in identifiable quantities. Besides homocoupling, a slight degradation of **2** to phenol was observed, a pathway that seems to be independent of the presence of **1** (Figure S6), and might be a noncatalytic photodegradation as was previously observed.^50^

Further insights into the conversion of **1** could be gathered through ^31^P NMR analysis of the reaction mixture after removal of the catalyst (Figure 4c), revealing distinct peaks in three regions. The expected peaks at approximately −5.95 and 29.6 ppm correspond to triphenylphosphine and its oxide, respectively. Interestingly, when TEA or KA were used, large peaks appeared at 21.8 ppm, replacing the unoxidized PPh_3_. Peaks in this region are indicative of the presence of tetraaryl phosphonium ions,^51^ suggesting an alternative reductive elimination step involving the phosphine ligand after the oxidative addition. While both Pd and Ni-catalyzed aryl halide-phosphine couplings have been reported with homogeneous catalysts,^52,53^ a dependence on the base was not observed as no base was required. Considering that only 10 mol % TPP was added, most of which was oxidized, the phosphonium side-product cannot solely account for the additional conversion of **1** (Figure S10). Thus, we hypothesize that other alternative reductive eliminations involving functionalities of the host materials are possible, although it is challenging to directly confirm this currently.

Regardless of the exact form of all side-products, it is necessary to differentiate between the effects of KA/TEA and KC/KP to gain a deeper understanding beyond the initial simplified p*K*_a_ description. Differences in base coordination could explain the reactivity patterns, as amines^14^ and acetate ions^15^ have previously been shown to coordinate to achieve selective SMC, whereas carbonate or phosphate complexes of Pd are generally uncommon. Consequently, competition between KA/TEA and TPP for the Pd centers could lead to the formation of phosphonium ions by pushing out TPP and the aryl group to enable base coordination, similar to how certain ligands promote reductive elimination through steric or electronic effects.^54,55^ In this scenario, the comparatively high excess of base (30:1 base to ligand) might instead act as a driving force. Other unidentified products could form through a similar mechanism. Subsequently, Pd sites that favor boronic acid homocoupling might form and be stabilized by the coordinating bases. Consequently, further analysis of the interplay between ligands and bases, and the resulting Pd state is necessary.

### 3.3 Palladium Activation and Phosphine-Free Operation

**Figure 5:**
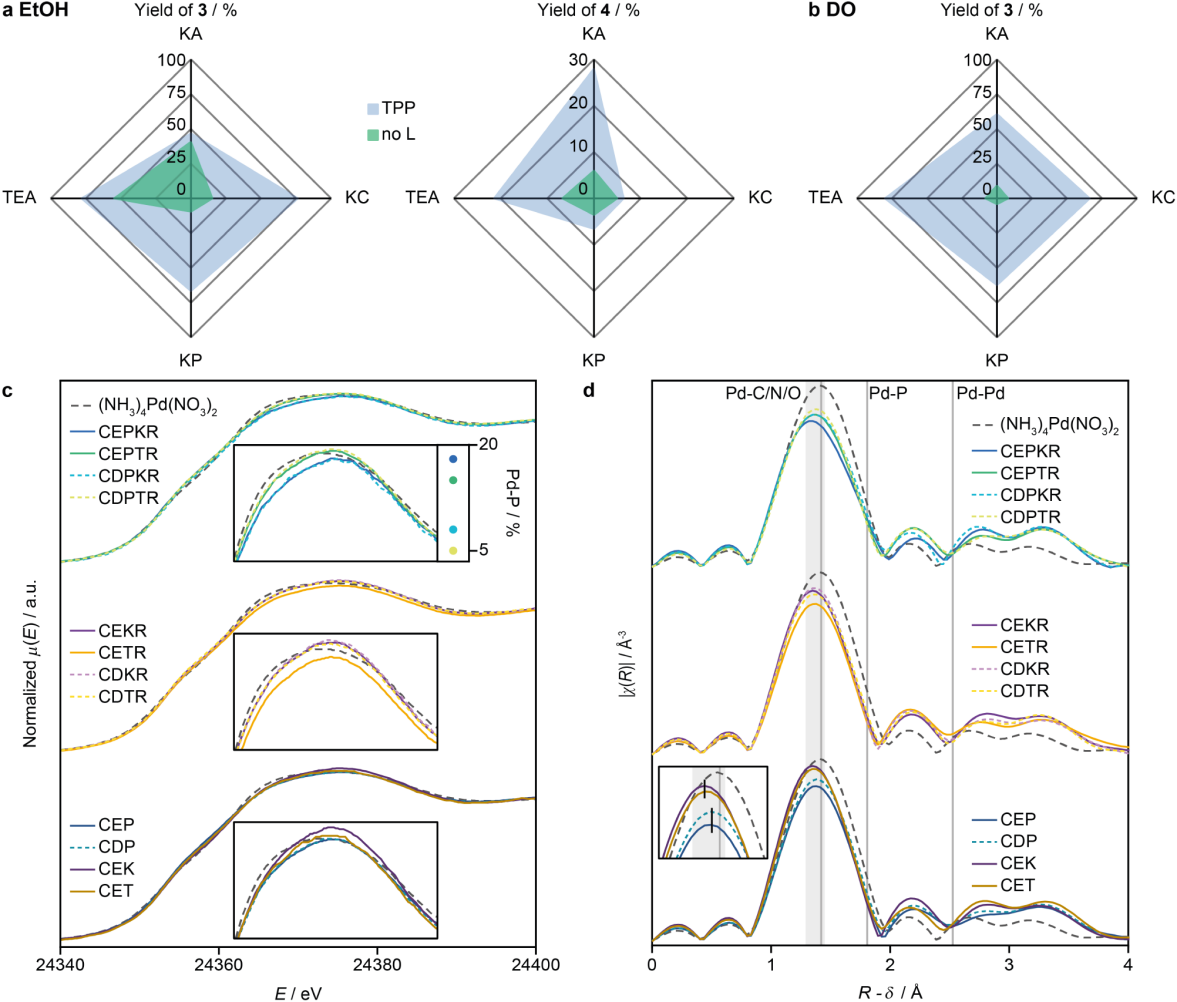
Effect of the absence of TPP on the yield of products **3** and **4** in ethanol (a) and dioxane (b). Reaction conditions: 0.1 mmol **1**, 1.5 equiv **2**,1 mol % Pd, 10 mol % TPP (if present), 3 equiv base, 1:0.3 organic to aqueous, 80 °C, 16 h. (c) In situ Pd *K*-edge XANES spectra of Pd_1_@C_3_N_4_ 2 h reaction time together with ex situ (NH_3_)_4_Pd(NO_3_)_2_. The insets show magnified spectra and the Pd–P contribution to the signal in the top case. (d) Fourier-transformed EXAFS spectra corresponding to the spectra in (c). The gray area highlights the region of Pd–C/N/O coordination while the gray lines indicate the average distance of Pd–N, Pd–P, and Pd–Pd in reference compounds (Figure S11). The inset compares changes in *R*-value between conditions exclusively containing bases or TPP. Abbreviations: C, catalyst (Pd_1_@C_3_N_4_); D, dioxane; E, ethanol; P, TPP; K, K_2_CO_3_; T, TEA; R, **1** and **2**, and water is present in all samples.

If KA/TEA coordination plays a crucial role, it could potentially enable phosphine-free SMC by fulfilling key functions of TPP. Indeed, intermediate yields were observed in ethanol in the absence of TPP using both KA or TEA (Figure 5a). Interestingly, homocoupling to biphenyl (**4**) was reduced to the levels observed with KC and KP combined with high selectivity for the conversion of **1** to **3** (Figure S9), suggesting that homocoupling follows alternative reductive eliminations or requires sites that are formed under the coexistence of both TPP and KA/TEA in ethanol. In contrast, comparable performance was not achieved when dioxane (Figure 5b) or acetonitrile were used as solvents (Table S8). The fact that appreciable yields of **3** could only be obtained in ethanol in the absence of the ligand, coupled with the lower resulting activity, indicates that KA/TEA could not entirely replace the phosphine ligand. Previous DFT simulations showed that part of the Pd activation in Pd_1_@C_3_N_4_ involved a step where the Pd atom is pulled to the top of the surface from its subsurface resting state, where it is inaccessible to reagents.^56^ This behavior was also calculated for TEA with a lower driving force, suggesting a “steric” activation to make the Pd atoms available for the reagents. The experimental results suggest that the ligand has additional roles for Pd activation that ethanol can replace to a lesser extent instead. This second contribution might instead be “electronic” complementing the steric effect.

XAS is an element-specific spectroscopic technique that is highly sensitive to the electronic and geometric structure of the target element.^57^ Accordingly, it can track changes to the probe atoms effectively and be used to identify the adsorption of molecules as well as reaction mechanisms, especially when conducted in in situ or operando modes.^57^ Thus, we performed in situ XAS experiments to investigate the changes in the oxidation state and coordination environment of the Pd centers to identify some of the changes hypothesized above. XANES measurements were conducted on the catalyst exposed to different combinations of ligand, solvent, base, and reagent for 2 h (Figure 5c). The in situ spectra of the catalyst most closely resemble the spectra of reference (NH_3_)_4_Pd(NO_3_)_2_ measured ex situ (Figures 5c and 2d), indicating small changes in the nominal Pd(II) oxidation state or coordination of the metal with the carrier. Instead, variations in white-line intensity suggest that partial reduction is achieved to varying extents by the different reagents. Under full reaction conditions, a stronger intensity decrease is observable using KC compared to TEA, independent of solvent which seems to have little effect. XANES linear combination analysis (LCA) shows pronounced Pd–P contribution in EtOH and dioxane (Table S3). These contributions are lower in the presence of TEA, agreeing with the competitive coordination of TPP and TEA.

Agreeing with the reactivity trends in ligand-free conditions, the strongest partial reduction is achieved when TEA and EtOH are combined, whereas changes among the other three combinations are more minute. Control experiments without the reagents were carried out to further probe the activation step and remove interference from the reagents. They confirm that TPP addition has the highest influence on the white-line intensity and leads to the strongest partial reduction, followed by TEA/EtOH.

Similar to the XANES analysis, extended X-ray absorption fine structure (EXAFS) analysis (Figure 5d) show high similitude to the Pd(II) reference, albeit with shifts in the Gaussian peak belonging to the Pd–N bonds toward slightly lower *R* values. Such deformations are likely due to shielding originating from a change in the coordination environment, providing evidence for the coordination of the different components.^57^ EXAFS fitting reveals average first-shell coordination numbers between 2.9 and 3.5 with second-period elements (Table S4). The fitting of the second shell was hampered by insufficient signal quality. Nonetheless, occupation of the second shell can be inferred, such as Pd–P via TPP coordination as evidenced by XANES fitting. Other options include Br, EtOH, TEA, etc. Averaging of various effects and all possible intermediate structures of the catalytic cycle is another challenge in addition to signal quality to fully identify the Pd environment. Consequently, determining the structure of reaction intermediates and transient states is not yet possible, except if they can be mimicked through a stepwise approach such as the one used herein for the initiation step.

### 3.4 Kinetics of the Coupling over Pd_1_@C_3_N_4_

**Figure 6:**
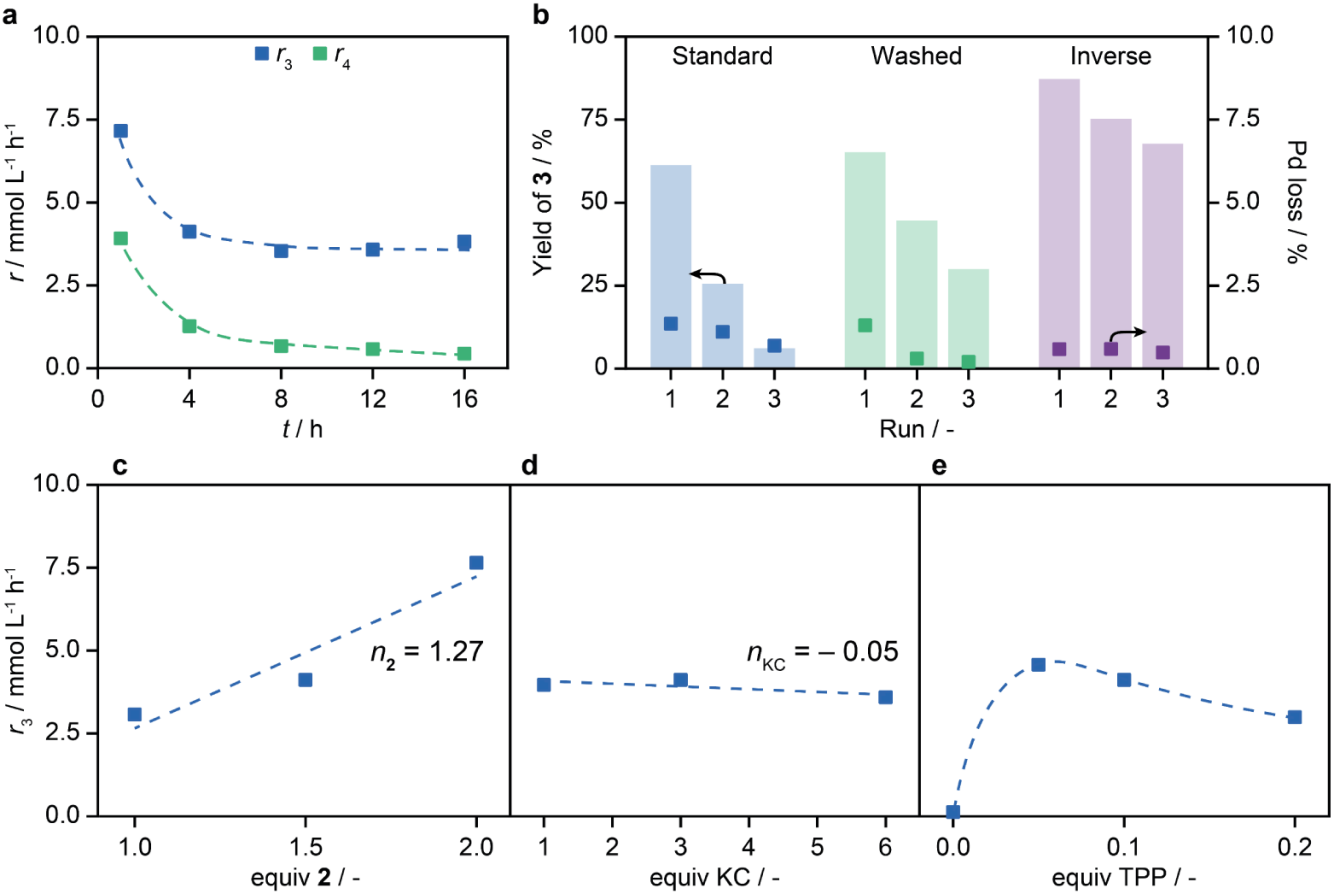
(a) Temporal evolution of the formation rate of products **3** and **4**. Reaction conditions: 0.1 mmol **1**, 1.5 equiv **2**,1 mol % Pd, 10 mol % TPP, 3 equiv KC, 1:0.3 dioxane to water, 80 °C. (b) Recycle tests of the catalyst under three different conditions indicating the Pd loss after every run. Standard conditions used in (a). Washed: the catalyst was washed with water after every run. Inverse: 0.3:1 dioxane to water ratio used instead. (c–e) Formation rates of product **3** after 4 h varying the equivalents of **2**, TPP, and KC, respectively, under conditions otherwise identical to (a). *n*_*j*_ designates the reaction order of reactant.

Following the mechanistic implications of the different components, their influence on the kinetics, which is currently lacking in SAC literature, was investigated under similar conditions to those described above. The KC dioxane system was chosen for all kinetic investigations to distinguish the individual impacts and prevent any previously discussed cross-effects. The observed behavior aligns with the homocoupling hypothesis stated for KC and KP, where biphenyl formation is related to the initial presence of O_2_ or a more partially oxidized Pd(II) species, as its formation rate peaks during the initial stages of the reaction after which it approaches zero (Figure 6a). A parallel decrease in activity was also observed for the main product, where its formation rate plateaued after the first 4 h. This pattern persists even when varying the equivalents of **2** (Figure S11). Accordingly, 4 h was chosen as a suitable cutoff time for further kinetic analysis while ensuring low yields (<30%) and more significant quantification.

The observed decrease in activity could stem from several factors, including catalyst restructuring, loss, or blockage of catalytically active sites via, for example, the deposition of inorganic salts. Verification of the first hypothesis is challenging using current methods as XAS, one of the most sensitive methods, cannot yet be used to identify transient species in the cycle as mentioned in Section 3.3. Additionally, palladium leaching would instead be expected to lead to an initial increase in activity, often as an induction period, and may thus be ruled out. Several observations suggest potential blockage of active sites. First, an increase in catalyst mass was noted postreaction, especially when KC and KP were used. Second, STEM analysis revealed the deposition of crystallites (Figure S12), likely inorganic salts such as KBr or KHCO_3_ which have lower solubility than the KC^58,59^ (K_2_HPO_4_ could form from KP instead). Lastly, changes in the textural properties of the catalyst were observed, with a granulated and less fluffy appearance coupled with a faster settling time after agitation or adhesion to the walls of the reaction vessel after the reaction (Figure S12). Consequently, the catalyst showed strong deactivation upon reuse, which cannot be related to the leaching (Figure 6b). The fouling caused by salt deposition may be reversible or preventable through further reaction environment optimization or the application of appropriate washing steps.^19^ First, a simple washing step employing deionized water was tried, which improved the reusability (Figure 6b). While washing partly restores some catalyst properties, such as its ability to be dispersed (Figure S12), the salt deposition, granulation, and adhesion problems upon reuse remain. Inversion of the solvent ratio again showed to be beneficial as the reusability increased further (Figure 6b) while seemingly maintaining the textural properties (Figure S12). Further optimization is likely to improve the reusability further, highlighting the need for dedicated regeneration studies.^19^

Kinetic analysis reveals the reaction starts near-first order in **2** (Figures 6c and S11), indicating that the transmetalation involving a boron species is rate-limiting, which agrees with a simplified DFT prediction for Pd_1_@C_3_N_4_ identifying the same rate-limiting step.^7^ Importantly, this observation contrasts with the findings of simulations of the SMC over other Pd SACs^8,60^ as well as experimental studies with some metal complexes^61,62^ that identified oxidative addition as rate-limiting. In rate-limiting transmetalation, the boronic acid order was observed to highly depend on its ratio to the base, with positive orders observed when using stronger bases in excess,^31,63^ conditions which are met here. Conversely, the initial reaction rate appears mostly independent of the base equivalents, showing a slight negative, near-zeroth order (Figure 6d), corroborating that the base does not participate in the rate-limiting step. These findings align with the existence of a fast equilibrium between the boronic acid-hydroxide and the boronate,^34^ and are consistent with prior kinetic results of non-SACs, which showed that the initial rate only weakly depends on the base amount and that larger base excesses can retard the rate due to an excess of hydroxide formation and ensuing active site blockage.^62,63^ Similar to the behavior expected for homogeneous palladium complexes, an optimal ligand-to-palladium ratio is observed at around 0.05 equiv, with a sharp initial increase followed by a slow decrease in activity measured (Figure 6e). Overbinding of the ligand can be expected to decelerate the reaction until enough of it is oxidized to triphenylphosphine oxide, which does not promote the catalytic reaction (Table S9). Considering the previous discussion, the optimum amount will heavily depend on the nature of the base used, as well as the amount of oxygen initially present. Overall, these trends qualitatively mirror those observed for some Pd-complexes with different thresholds and optima as well as some specificities such as an initial deactivation, emphasizing the nuanced interplay of reaction components with the catalyst in governing the catalyst kinetics.

The insights gained from studying Pd_1_@C_3_N_4_ could pave the way for newer systems that address some of the limitations of the initial SAC system employed for the SMC. Designing catalysts with amphiphilic surfaces (e.g., an inherently amphiphilic support) could extend the compatibility with a broader range of solvents. Alternatively, strategies to shift wetting behaviors via surface functionalization of otherwise hydrophilic systems might be necessary to facilitate process integration of the coupling step when the solvent choice is limited. Exploring different ligands could also be promising for increasing base compatibility by using more stable and strongly coordinating phosphine ligands. These ligands could sterically or electronically prevent base coordination to the Pd centers and have a minimized degradation if their oxidation is disfavored.^64^ For instance, replacing TPP with RuPhos shows potential for suppressing side reactions like homocoupling and phosphonium formation while achieving comparable yields (Figure S13 and Table S9). Integrating basic properties in the support could be a strategy to eliminate the need for external bases. For example, Pd/MgO has shown promising first results in polar solvents (Table S10). Moreover, to avoid the need for phosphine ligands in nonreducing conditions, the Pd sites should initially be present in a more reduced state and coordinatively available. Metal oxide-based systems might be able to create such sites as the Pd atom is hypothesized to be located between bridging oxygens, with the Pd atoms accessible at the surface.^8,12^ Exploring these concepts further, will be the focus of future studies, where essential design parameters will be the ability of the carrier to stabilize reduced and coordinatively available Pd centers. Nevertheless, catalysts should in all cases be tested under appropriate conditions, their kinetics measured, and their optimal conditions found to confirm such behaviors experimentally. To our knowledge, observations such as the selectivity loss caused by TEA–TPP combinations have not been reported yet. As different SACs may exhibit distinct behavior, relying on theoretical simulations that might not model the surfaces accurately or treating them as drop-in solutions assuming identical working principles compared to existing catalysts, will not capture all features.

## 4 Conclusion

This study investigated the mechanism of the heterogeneously catalyzed Suzuki–Miyaura coupling using Pd_1_@C_3_N_4_ as a model single-atom system, exploring reactivity patterns across solvents and bases with diverse properties. The choice of solvent-base pair emerged as a crucial factor for ensuring optimal catalyst surface wetting, maximizing the creation of interfaces within the triphasic reaction system, and preventing catalyst fouling via base deposition. This requires balancing the base strength, solubility, solvent polarity, and catalyst surface tension. These previously unexplored aspects have important implications not only for Pd_1_@C_3_N_4_ but also heterogeneous systems in general. Concerning the reaction mechanism and kinetics, solvent and base selection were also highly influential. Bases with coordinating abilities facilitated the formation of active sites that catalyze side reactions such as boronic acid homocoupling, by competitively coordinating with the phosphine ligand. Conversely, these same coordinating bases enabled phosphine-free pathways in solvents providing sufficient reducing conditions, offering design principles for ligand-free operation. In situ X-ray absorption spectroscopy identified changes in the electronic and coordinating structure of the Pd sites induced by the ligand, solvent, and base, supporting the observed selectivity and activity patterns. Kinetic tests identified transmetalation as rate-limiting, confirming previous theoretical hypotheses for Pd_1_@C_3_N_4_ while contrasting with predictions for other SACs. These insights highlight the current challenges in developing heterogeneous single-atom catalysts for coupling applications and emphasize the importance of a deeper understanding of the mechanistic and kinetic complexities in this intricate and variable reaction environment.

## Data Availability

The data generated in this study is available on Zenodo: 10.5281/zenodo.11073240.

## Supporting Information Available

Supporting characterization, catalytic, and analytical data; methods, measurements, and supporting figures and tables (PDF)
