# Supplementary material for: Understanding and Controlling Reactivity Patterns of Pd1@C3N4-Catalyzed Suzuki–Miyaura Couplings
Source: ACS Catal. 2024 Aug 7;14(16):12635–46. doi: 10.1021/acscatal.4c03531 (PMC11334102; doi:10.1021/acscatal.4c03531)
Supplement: Supplementary file 1 — cs4c03531_si_001.pdf [file cs4c03531_si_001.pdf]

Supporting Information

Understanding and Controlling Reactivity Patterns of $Pd_1@C_3N_4$-Catalyzed Suzuki-Miyaura Couplings

M.E. Usteri,[a] *G. Giannakakis,*[a]* *A. Bugaev,*[b] *J. Pérez-Ramírez,*[a]* *S. Mitchell*[a]*

[a] Institute of Chemical and Bioengineering, Department of Chemistry and Applied Biosciences, ETH Zurich, Vladimir-Prelog-Weg 1, 8093 Zurich, Switzerland

[b] Paul Scherrer Institute, Forschungsstrasse 111, 5232 Villigen, Switzerland

* E-mail: ggiannakakis@chem.ethz.ch; jpr@chem.ethz.ch; sharon.mitchell@chem.ethz.ch

Table of Contents

Supplementary Methods 1
Supplementary Note 2
Supplementary Tables 3
Supplementary Figures 13
References 26

Supplementary Methods

The synthesis of other carrier materials was based on previously reported protocols.

Nitrogen-doped carbon (NC):[1] Activated carbon (Norit Rox 0.8) was mixed with concentrated nitric acid (1 g per 20 mL) and refluxed at 80°C for 16 h. After pouring the suspension into ice-cooled deionized water (DIW), the powder was filtered off, thoroughly washed with DIW, and dried overnight (80°C). The acid-treated activated carbon was mixed with dicyandiamide (1:3 mass ratio) and ground in a mortar. The resulting solid mixture was carbonized *via* a two-step process (450°C for 3 h, 600°C for 2 h, 5°C h^{-1} temperature ramp) in flowing nitrogen, yielding nitrogen-doped carbon.

Polyaniline-derived carbon (PAC):[2] A precooled solution of ammonium persulfate in DIW (4°C, 0.25 mol, 100 mL) was mixed with a precooled solution of aniline (0.5 mol) and phytic acid (0.1 mol) in DIW (100 mL) and stirred for 1 h in an ice bath. After polymerization for 24 h at room temperature, the solid was thoroughly washed with DIW until reaching a neutral pH and dried overnight *in vacuo* (80°C). The polymer was then pre-carbonized in static nitrogen at 400°C (1 h, 2°C min^{-1} temperature ramp), mixed and ball-milled (20 Hz, 15 min) with a mass-based three-fold amount of KOH and carbonized in static nitrogen at 800°C (1 h, 5°C min^{-1} temperature ramp). Finally, all residual KOH was washed away with DIW to achieve a neutral pH and the carbon material was vacuum-dried.

Sulphur-doped carbon (SC):[3] 1,3,5-tris(4-bromophenyl)benzene (5 mmol), $^{i}Pr_2Net$ (40 mmol), and dry 1,4-dioxane (20 mL) were mixed in an oven-dried round-bottom flask and evacuated and backfilled with nitrogen (three cycles). After the addition of $Pd_2(dba)_3$, Xantphos (1 mmol), and thiophenol (30 mmol), the mixture was degassed twice more and refluxed for 24 h. The reaction mixture was then filtered, and concentrated under reduced pressure and the product was purified by flash column chromatography on silica gel with *n*-hexane: DCM (9:1 to 7:3). After further purification by slow diffusion of *n*-hexane in DCM, 1,3,5-tris(4-phenylthiophenyl)benzene was obtained. In a glovebox, LiHMDS (6 mmol) and 1,3,5-tris(4-phenylthiophenyl)benzene (1 mmol) were dissolved in an anhydrous o-xylenes-diphenyl ether mixture (1:1, 200 mL). After complete dissolution, thiophenol (0.1 mmol) and Pd-Singacycle-A1 (0.02 mmol) were added, and the flask was sealed and placed in a preheated oil bath (80°C) outside the glovebox. After 20 h without stirring, the solution was cooled, the solid separated from the solution and thoroughly washed (2 x toluene, 2 x N,N-dimethylformamide,

2 x water, 2 x ethanol, 2 x dichloromethane, and 2 x hexane). Finally, the solid was dried at 50°C under flowing N_2 overnight.

Metal Introduction: The procedure for Pd introduction followed that of $Pd_1@C_3N_4$, with the following exceptions. For SC the annealing temperature was lowered to 120°C and the time increased to 12 h, and for commercially available MgO no microwave treatment was applied.

Supplementary Note

Note S1 | Comment on contact angle measurement. The thermal exfoliation-based methodology used for catalyst preparation in this study precluded the formation of extended flat surfaces. Instead, measurements were attempted by pressing the catalyst powder into disc-shaped pellets. The resulting surfaces were likely to contain considerable roughness potentially leading to either Cassie-Baxter or Wenzel behaviours. Accordingly, a comparison between different carriers would not have been possible due to variable roughness contributions and instead limited to the comparison of different solvents on one carrier. Unfortunately, the roughness combined with the intrinsic porosity of the carrier and the volatility of the solvents made the formation of a 10 μL drop on top of the pellet impossible as the liquid would simultaneously evaporate and be absorbed by capillary forces.

Supplementary Tables

Table S1. Overview of reagents used, including CAS number and supplier.

Name	CAS	Supplier
dicyandiamide	461-58-5	Sigma-Aldrich
Norit Rox 0.8	7440-44-0	Cabot Corp.
nitric acid (>65%)	7697-3732	Sigma-Aldrich
aniline	62-53-3	Acros
ammonium persulfate	7727-54-0	Sigma-Aldrich
phytic acid	83-86-3	Tokyo Chemical Industry
potassium hydroxide	1310-58-3	Fisher Chemical
LiHMDS	4039-32-1	Sigma-Aldrich
1,3,5-Tris(4-bromophenyl)benzene	7511-49-1	Sigma-Aldrich
Xantphos	161265-03-8	Sigma-Aldrich
$Pd_2(dba)_3$	51364-51-3	Fluorochem
$^iPr_2NEt$	7087-68-5	Sigma-Aldrich
thiophenol	108-98-5	Acros
diphenyl ether	101-84-8	abcr
Pd Singacycle A1	930796-10-4	Tokyo Chemical Industry
MgO	1309-48-4	Strem
$(NH_3)_4Pd(NO_3)_2$ (10 wt% in water)	13601-08-6	Sigma-Aldrich
4-bromotoluene	106-38-7	abcr
phenylboronic acid pinacol ester	24388-23-6	Appollo Scientific
triphenylphosphine	603-35-0	Sigma-Aldrich
mesitylene	108-67-8	Thermo Scientific
potassium acetate	127-08-2	Sigma-Aldrich
potassium carbonate	584-08-7	Sigma-Aldrich
potassium phosphate	7778-53-2	Sigma-Aldrich
triethyl amine	121-44-8	Merck
ethanol	64-17-5	Supelco
acetonitrile (HPLC grade)	75-05-8	Sigma-Aldrich
dioxane	123-91-1	Fisher Scientific
N,N-dimethylformamide	68-12-2	Sigma-Aldrich
dichloromethane	75-09-2	Sigma-Aldrich
hexane	110-54-3	Sigma-Aldrich
o-xylene	95-47-6	Thermo Scientific
toluene	108-88-3	Fisher Scientific
acetonitrile-d3	2206-26-0	Eurisotope
D_2O	7789-20-0	Appollo Scientific

Table S2. Quantification of Pd content of the different as-prepared catalysts used in this study.

Catalyst	Pd content / wt%[a]
$Pd_1@C_3N_4$	0.52
Pd/NC	0.45
Pd/PAC	0.48
Pd/SC	0.17
Pd/MgO	0.98

[a] Determined by ICP-OES.

Table S3. XANES fitting parameters from in situ measurements of $Pd_1@C_3N_4$ in non-interacting toluene as a reference and in solutions containing distinct solvent, base, ligand, and reactant combinations. The fit was performed for the normalized XANES spectra in the 24335-4405 eV region. The example of the fit is shown in **Figure S1**.

System[a]	Fitting model[b]	Contribution / %			R-factor / %
		Pd-P[c]	Pd(0)[d]	Pd(II)[e]	
CEPKR	1	-	5.0	95.0	0.26
	2	18.0	0.0	82.0	0.16
CEPTR	1	-	0.1	99.9	0.13
	2	8.0	0.0	92.0	0.11
CDPKR	1	-	2.7	97.3	0.26
	2	15.0	0.0	85.0	0.19
CDPTR	1	-	0.0	100	0.12
	2	5.0	0.0	95.0	0.11
CEKR	1	-	0.0	100	0.11
CETR	1	-	0.2	99.8	0.39
CDKR	1	-	0.0	100	0.12
CDTR	1	-	0.0	100	0.12
CEP	1	-	2.1	97.9	0.16
	2	11.0	0.0	89.0	0.12
CDP	1	-	0.0	100	0.16
	2	8.0	0.0	92.0	0.14
CEK	1	-	0.0	100	0.15
CET	1	-	0.0	100	0.17

[a] The codes indicate the reagents present in each measurement: Catalyst (C), solvent (E - ethanol, D - 1,4-dioxane), base (K - K_2CO_3 or T - NEt_3, ligand: P - PPh_3), and reactants (R - p-bromotoluene and phenylboronic acid piacol ester). [b] Fitting model 1 considers only Pd(II) and Pd(0) components while model 2 additionally includes a Pd-P contribution. The following references were used: [c] $Pd(TPP)_4$, [d] Pd foil, [e] $(NH_3)_4Pd(NO_3)_2$.

Table S4. EXAFS fitting parameters from *in situ* measurements of $Pd_1@C_3N_4$ in non-interacting toluene as a reference and in solutions containing distinct solvent, base, ligand, and reactant combinations. The fit was performed in *R*-space for 1–2.5 Å region using $k^{1,2,3}$-weighted data, Fourier-transformed in 1.7–8 $Å^{-1}$ *k*-range. All data were fitted by a single Pd–O path, with variable interatomic distance (*R*), coordination number (*N*), and Debye-Waller parameter (σ^2). The zero-potential correction (ΔE_0) was fixed to 0. An example of the fit is shown in **Figure S1.**

System[a]	*R* / Å	Coordination number / -	σ^2 / $Å^2$	*R*-factor / %
CEPKR	1.97(2)	3.4(7)	0.005(4)	1.45
CEPTR	1.97(2)	3.1(6)	0.002(4)	1.71
CDPKR	1.96(2)	2.9(5)	-0.001(3)	1.30
CDPTR	1.96(2)	3.1(7)	0.002(4)	1.90
CEKR	1.95(2)	3.3(8)	0.003(4)	1.95
CETR	1.96(3)	2.8(7)	0.002(5)	2.69
CDKR	1.95(2)	3.4(8)	0.003(4)	1.86
CDTR	1.96(2)	3.3(7)	0.003(4)	1.74
CEP	1.97(2)	3.2(5)	0.002(3)	1.03
CDP	1.97(2)	3.1(6)	0.001(3)	1.33
CEK	1.96(2)	3.5(7)	0.003(4)	1.52
CET	1.95(2)	3.3(8)	0.002(4)	2.09

[a] The codes indicate the reagents present in each measurement: Catalyst (C), solvent (E - ethanol, D - 1,4-dioxane), base (K - K_2CO_3 or T - NEt_3, ligand: P - PPh_3), and reactants (R - p-bromotoluene and phenylboronic acid piacol ester).

Table S5. Performance of $Pd_1@C_3N_4$ in the SMC under different reaction environments.

Reaction[a]	Solvent[b]	Base[c]	Conversion **1** / %	Yield **3** / %[d]	Yield **4** / %[e]	Pd loss / wt%	Leached Pd / ppm
1	EtOH	KA	74	47	28	0.50	0.43
2	EtOH	TEA	100	78	21	1.09	0.93
3	EtOH	KC	81	76	6	0.98	0.83
4	EtOH	KP	71	67	7	1.09	0.93
5	EtOH	none	10	2	5	n.d.	n.d.
6	MeCN	KA	55	38	20	0.51	0.44
7	MeCN	TEA	96	76	16	0.36	0.31
8	MeCN	KC	80	78	7	0.94	0.80
9	MeCN	KP	55	52	3	0.71	0.60
10	MeCN	none	11	2	6	n.d.	n.d.
11	dioxane	KA	77	61	18	0.60	0.51
12	dioxane	TEA	92	80	14	0.56	0.48
13	dioxane	KC	69	66	5	0.42	0.36
14	dioxane	KP	65	62	3	0.78	0.66
15	dioxane	None	4	2	6	n.d.	n.d.
16	toluene	KA	6	4	1	n.d.	n.d.
17	toluene	TEA	13	11	2	n.d.	n.d.
18	toluene	KC	11	7	1	n.d.	n.d.
19	toluene	KP	5	3	1	n.d.	n.d.
20	toluene	none	3	1	1	n.d.	n.d.

[a] Reaction conditions: 0.1 mmol 4-bromotoluene (**1**, *p*TolBr), 1.5 equiv phenylboronic acid pinacole ester (**2**, PhBPin), 1 mol% Pd, 10 mol% triphenylphosphine (TPP), 3 equiv base, 1 mL solvent, 0.3 mL water, 80°C, 16 h. [b] EtOH: ethanol, MeCN: acetonitrile. KA: potassium acetate, TEA: triethylamine, KC: potassium carbonate, KP: potassium phosphate. [d] Gas chromatography (GC) yield with respect to **1**. [e] GC yield with respect to **2**.

Table S6. Performance evaluation of further Pd-SACs in the SMC.

Reaction[a]	Solvent	Base	Catalyst	Conversion **1** / %	Yield **3** / %	Yield **4** / %
21	toluene	KA	Pd/NC[b]	3	2	1
22	toluene	TEA	Pd/NC	4	1	0
23	toluene	KC	Pd/NC	4	1	0
24	toluene	KP	Pd/NC	5	1	0
25	toluene	none	Pd/NC	5	1	0
26	EtOH	TEA	Pd/NC	8	4	0
27	MeCN	TEA	Pd/NC	1	3	3
28	dioxane	TEA	Pd/NC	3	1	0
29	toluene	KA	Pd/PAC[c]	3	1	0
30	toluene	TEA	Pd/PAC	5	2	0
31	toluene	KC	Pd/PAC	5	1	0
32	toluene	KP	Pd/PAC	6	1	0
33	toluene	none	Pd/PAC	5	1	0
34	EtOH	TEA	Pd/PAC	7	2	0
35	MeCN	TEA	Pd/PAC	7	2	0
36	dioxane	TEA	Pd/PAC	5	1	0
37	toluene	KC	Pd/SC[d]	63	60	10

[a] Reaction conditions: 0.1 mmol *p*TolBr (**1**), 1.5 equiv PhBPin (**2**), 1 mol% Pd, 10 mol% TPP, 3 equiv base, 1 mL solvent, 0.3 mL water, 80°C, 16 h. [b] Nitrogen-doped carbon. [c] Polyaniline-derived carbon. [d] Sulfur-doped carbon, 0.33 mol% Pd was used instead.

Table S7. Performance of $Pd_1@C_3N_4$ in the SMC with inverted solvent-to-water ratio.

Reaction[a]	Solvent	Base	Conversion **1** / %	Yield **3** / %	Yield **4** / %
38	EtOH	KA	75	53	11
39	EtOH	TEA	100	86	10
40	EtOH	KC	93	90	4
41	EtOH	KP	100	97	7
42	MeCN	KA	77	64	19
43	MeCN	TEA	100	93	8
44	MeCN	KC	100	97	9
45	MeCN	KP	92	91	9

[a] Reaction conditions: 0.1 mmol *p*TolBr (**1**), 1.5 equiv PhBpin (**2**),1 mol% Pd, 10 mol% TPP, 3 equiv base, 0.3 mL solvent, 1 mL water, 80°C, 16 h.

Table S8. Performance of $Pd_1@C_3N_4$ in the SMC in the absence of phosphine ligand.

Reaction[a]	Solvent	Base	Conversion **1** / %	Yield **3** / %	Yield **4** / %
46	EtOH	KA	41	41	6
47	EtOH	TEA	55	55	7
48	EtOH	KC	26	25	5
49	EtOH	KP	13	10	4
50	EtOH	none	4	1	2
51	MeCN	KA	16	16	16
52	MeCN	TEA	17	16	8
53	MeCN	KC	5	3	4
54	MeCN	KP	5	4	5
55	MeCN	none	2	1	2
56	dioxane	KA	10	9	10
57	dioxane	TEA	10	8	5
58	dioxane	KC	8	8	4
59	dioxane	KP	5	4	3
60	dioxane	none	2	2	2
61	toluene	KA	3	1	2
62	toluene	TEA	4	1	2
63	toluene	KC	2	1	1
64	toluene	KP	3	1	0.0
65	toluene	none	3	1	3

[a] Reaction conditions: 0.1 mmol *p*TolBr (**1**), 1.5 equiv PhBpin (**2**), 1 mol% Pd, 3 equiv base, 1 mL solvent, 0.3 mL water, 80°C, 16 h.

Table S9. Performance of $Pd_1@C_3N_4$ in the SMC with triphenylphosphine oxide (TPPO) or 2-dicyclohexylphosphino-2′,6′-diisopropoxybiphenyl (RuPhos) instead of TPP.

Reaction[a]	Solvent	Ligand	Base	Conversion **1** / %	Yield **3** / %	Yield **4** / %
66	dioxane	TPPO	KA	6	3	5
67	dioxane	TPPO	TEA	5	3	4
68	dioxane	TPPO	KC	4	2	4
69	dioxane	TPPO	KP	11	7	6
70	EtOH	RuPhos	KA	31	28	5
71	EtOH	RuPhos	TEA	74	70	4
72	EtOH	RuPhos	KC	54	50	5
73	EtOH	RuPhos	KP	38	44	4
74	dioxane	RuPhos	KA	25	25	5
75	dioxane	RuPhos	TEA	48	47	8
76	dioxane	RuPhos	KC	55	54	9
77	dioxane	RuPhos	KP	12	12	7

[a] Reaction conditions: 0.1 mmol *p*TolBr (**1**), 1.5 equiv PhBPin (**2**), 1 mol% Pd, 10 mol% RuPhos, 3 equiv base, 1 mL solvent, 0.3 mL water, 80°C, 16 h.

Table S10. Preliminary performance of Pd/MgO in the SMC without adding an external base to the reaction mixture.

Reaction[a]	Solvent	Ligand	Conversion **1** / %	Yield **3** / %	Yield **4** / %
78	EtOH	TPP	96	84	39
79	EtOH	none	98	94	4
80	dioxane	TPP	84	80	8
81	dioxane	None	46	40	7
82	toluene	TPP	7	5	7
83	toluene	none	4	2	14

[a] Reaction conditions: 0.1 mmol *p*TolBr (**1**), 1.5 equiv PhBPin (**2**), 1 mol% Pd, 10 mol% TPP, 1 mL solvent, 0.3 mL water, 80°C, 16 h.

Supplementary Figures

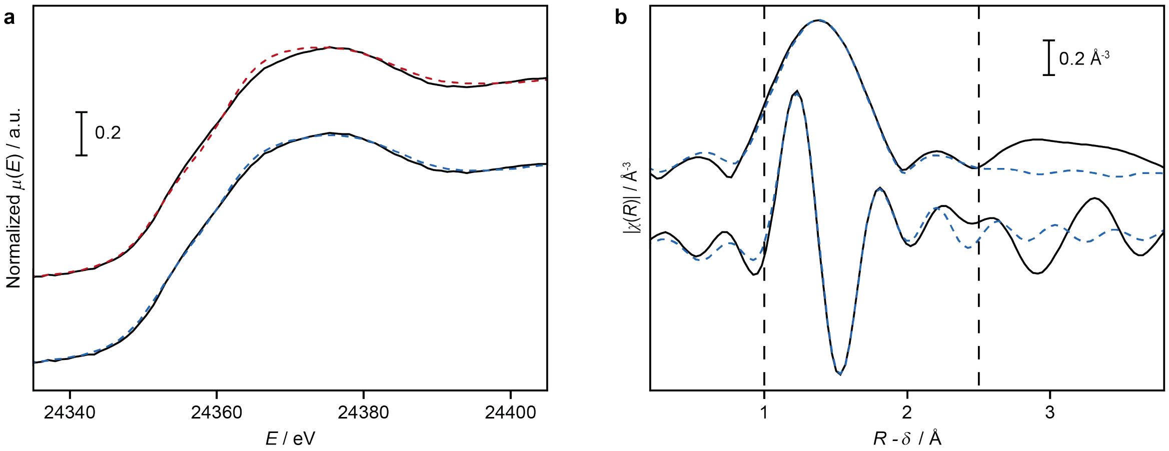

Figure S1. The results of (a) LCF of experimental XANES spectrum (solid lines) using only the two reference spectra of Pd foil and $(NH_3)_4Pd(NO_3)_2$ (dashed red, model 1) and with addition of $Pd(TPP)_4$ reference (dashed blue, model 2); and (b) EXAFS fit (dashed blue) of the experimental EXAFS spectrum (both amplitude and imaginary parts) of the *in situ* measurement of $Pd_1@C_3N_4$ under full reaction condition with KC as a base (corresponding to entries CEPKR in **Tables S3** and **S4**).

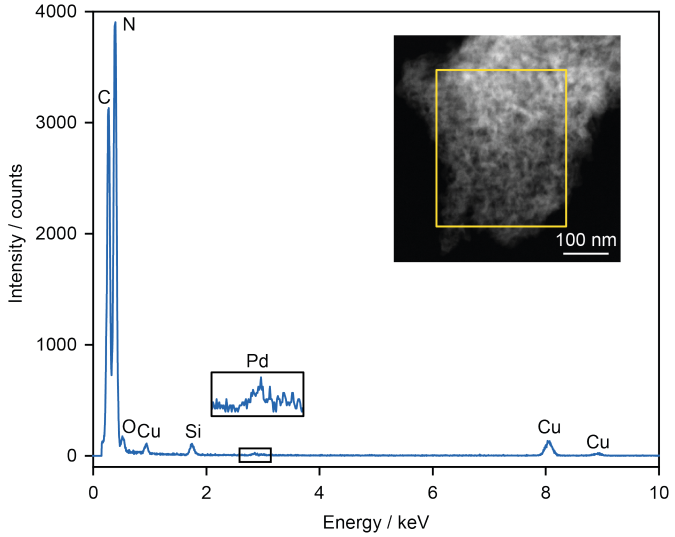

Figure S2. Energy-dispersive X-ray spectrum acquired from the sample region analyzed in the micrograph in **Figure 2c** with assigned peaks. The investigated portion of the sample is shown inset. The Cu signal originates from the sample holder and microscopy grid while Si originates from the detector.

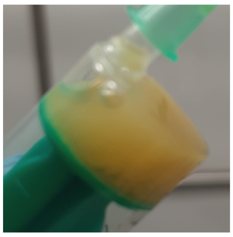

Figure S3. Photograph of the Pd/SC catalyst dispersion in the biphasic toluene/aqueous K_2CO_3 mixture (**Table S4**, entry 45).

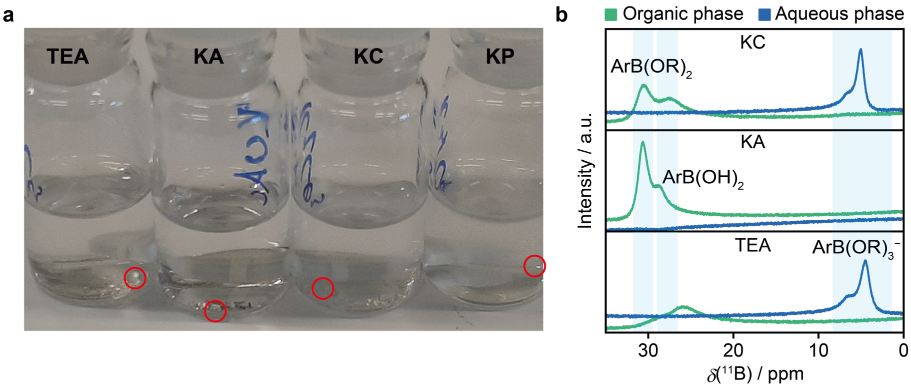

Figure S4. a Emulsions of the different aqueous base solutions with a MeCN solution containing all the reagents after 30 min of stirring. The red circles show organic droplets formed. **b** ^{11}B-NMR spectra of the reaction mixtures after 30 min of stirring without catalyst. Emulsions with KP could not be separated.

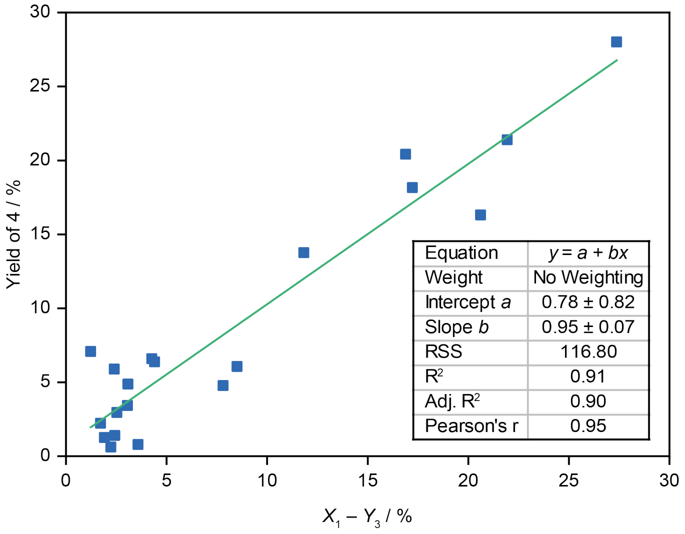

Figure S5. Linear fit and correlation analysis between the mass balance gap of **1** (expressed as the difference between the conversion of **1** X_1 and yield of **3** Y_3) and the yield of **4**. A linear fit was applied using the software OriginPro[4] and the statistical data is shown in the inset. A correlation parameter of 0.95 was obtained using Pearson's methods, showing a strong positive correlation.

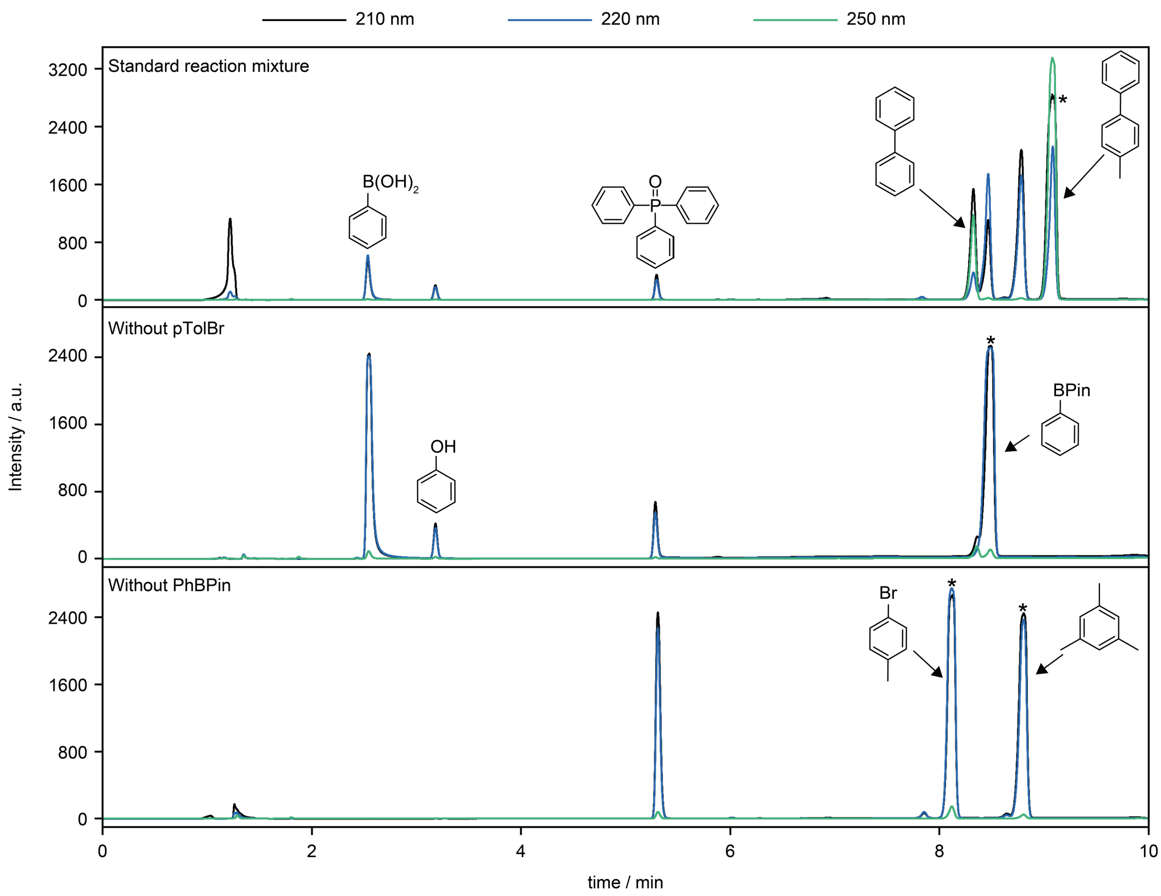

Figure S6. HPLC chromatograms of different reaction mixtures measured with a UV detector at three different wavelengths. Reaction conditions top chromatogram: 0.1 mmol **1**, 1.5 equiv **2**,1 mol% Pd, 10 mol% TPP, 3 equiv TEA, 1:0.3 organic to aqueous, 80°C, 16 h. Middle chromatogram: without **1**. Bottom chromatogram: without **2**. Peaks marked with * saturated the UV detector. Saturation was chosen to improve the signal-to-noise ratio of side products. Peaks were assigned by injecting references.

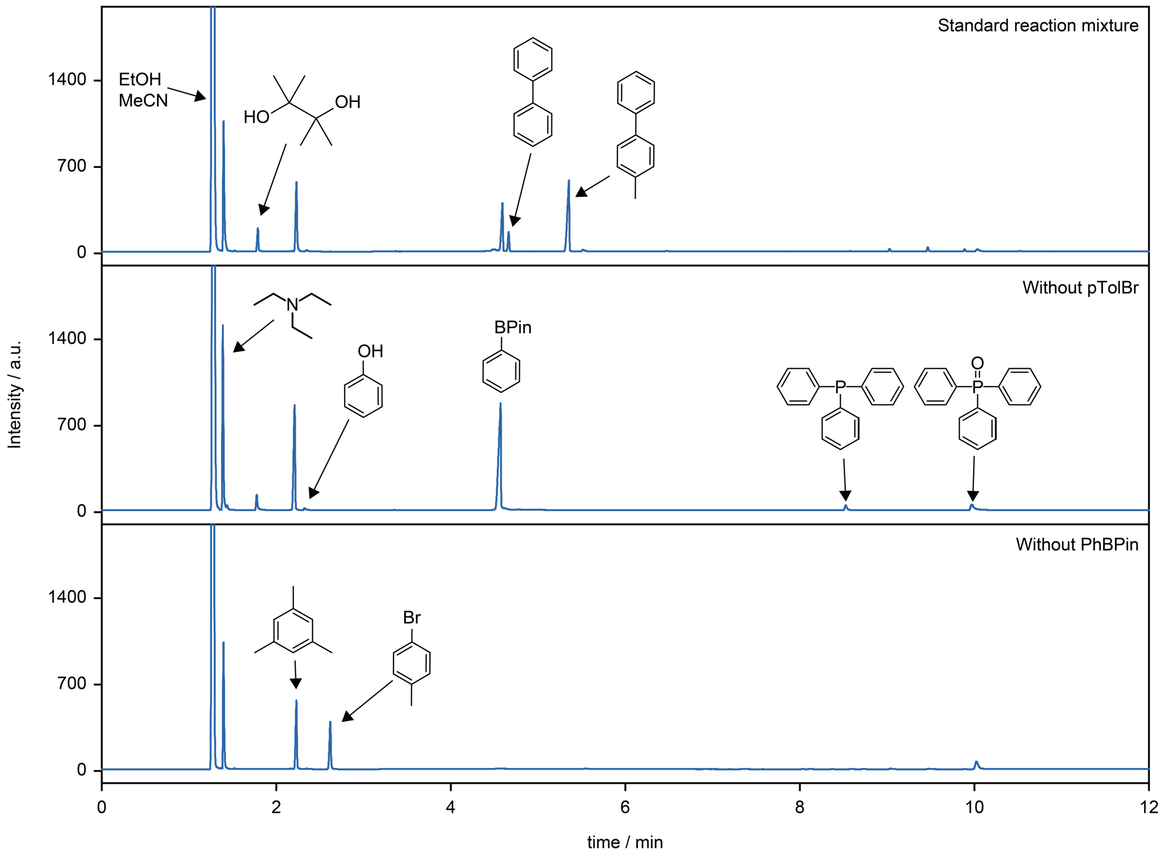

Figure S7. GC chromatograms of different reaction mixtures measured with an FID detector. Reaction conditions top chromatogram: 0.1 mmol **1**, 1.5 equiv **2**, 1 mol% Pd, 10 mol% TPP, 3 equiv TEA, 1:0.3 organic to aqueous, 80°C, 16 h. Middle chromatogram: without **1**. Bottom chromatogram: without **2**, 1 equiv TPP. Peaks were assigned by injecting references and with the help of a GC-MS.

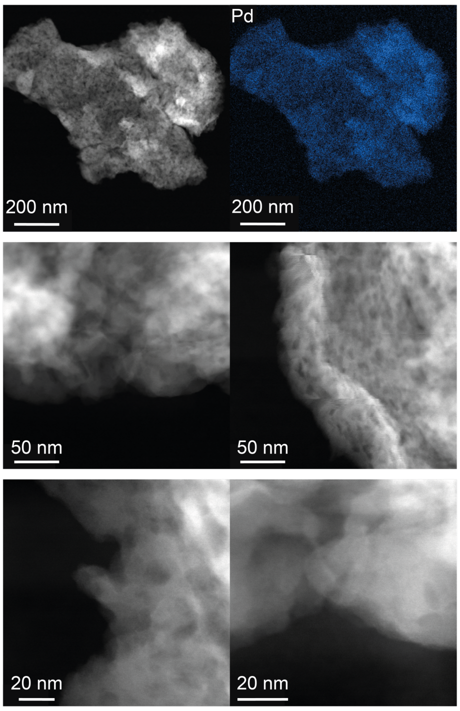

Figure S8. HAADF-STEM micrographs and EDX elemental mapping of used $Pd_1@C_3N_4$ catalysts. Reaction conditions for all samples: 0.1 mmol **1**, 1.5 equiv **2**,1 mol% Pd, 10 mol% TPP, 3 equiv TEA, 1:0.3 EtOH to water, 80°C, 16 h.

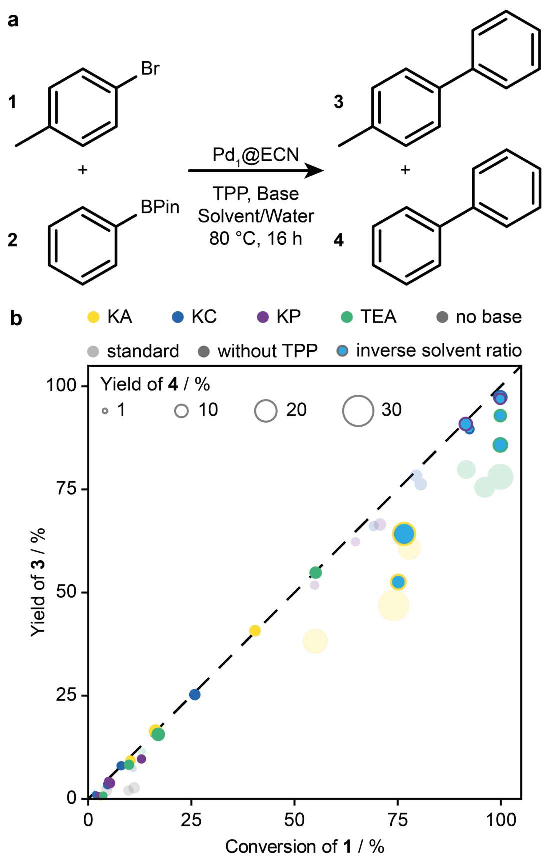

Figure S9. a Scheme of the Suzuki-Miyaura coupling of 4-bromotoluene **1** and phenylboronic acid pinacol ester **2** over $Pd_1@C_3N_4$. **b** Extended conversion-yield map including the standard reaction mixture and conditions without ligand and with inverted solvent ratio. The size of the circles represents the yield of the main side product biphenyl **4** based on the initial amount of **2**.

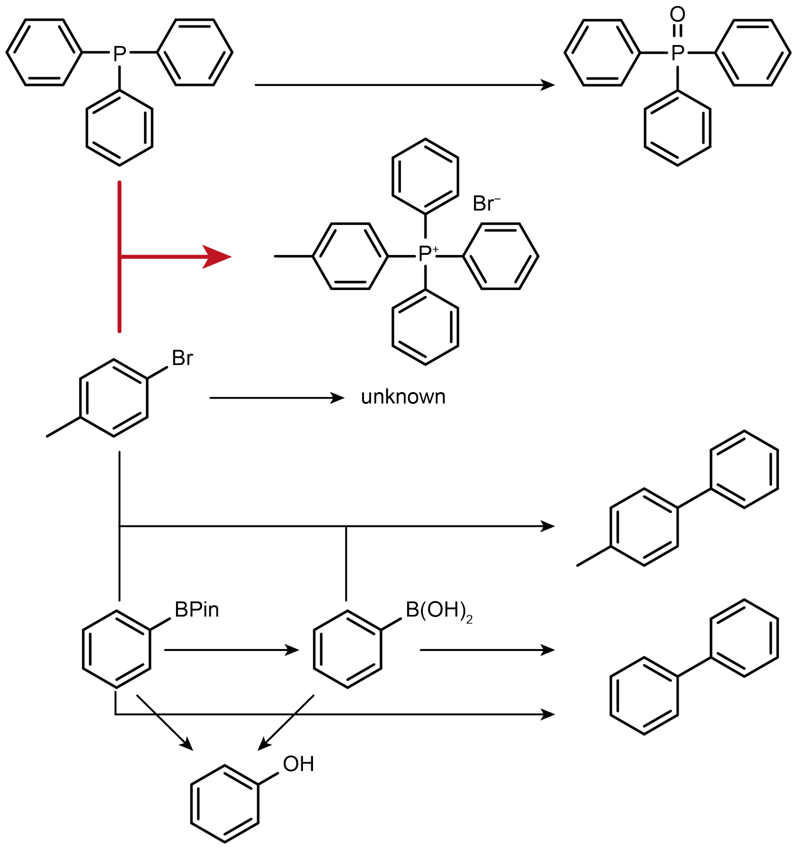

Figure S10. Simplified reaction network with all identified products. Based on the initial ratios, the yield of phosphonium (formed via the red arrow) cannot exceed 10% and is likely lower due to more than 50% oxidation of TPP. The presence of an unidentified product is inferred from the gap between conversion and yield, which exceeds 10%.

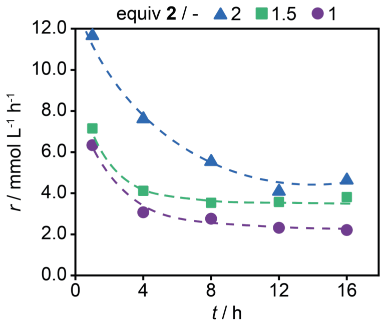

Figure S11. Temporal evolution of the formation rate of product **3** at different equivalents of reagent **2.** Reaction conditions: 0.1 mmol **1**, 1 mol% Pd, 10 mol% TPP, 3 equiv KC, 1:0.3 DO to water, 80°C, 16 h.

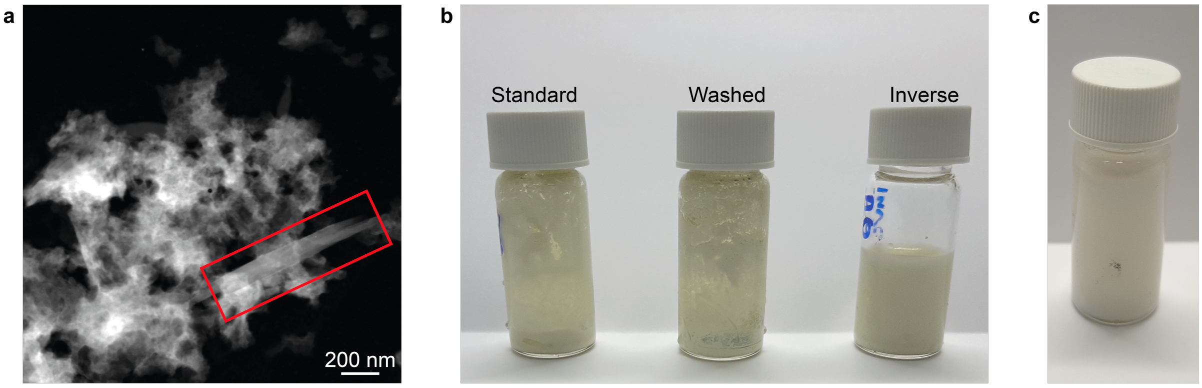

Figure S12. a HAADF-STEM micrograph of used $Pd_1@C_3N_4$ after 4 h in the SMC. The red box shows a salt crystal deposited on the catalyst particle in the case of KBr, K_2CO_3, or $KHCO_3$. Reaction conditions: 0.1 mmol **1**, 1.5 equiv **2**,1 mol% Pd, 10 mol% TPP, 3 equiv KC, 1:0.3 EtOH to water, 80°C, 4 h. **b** Photograph of the reaction mixture after the third reuse. Standard: Conditions used in a. Washed: The catalyst was washed with water after every run. Inverse: 0.3:1 dioxane to water ratio used instead. **c** Catalyst suspension after washing through stirring in deionized water for 40 min.

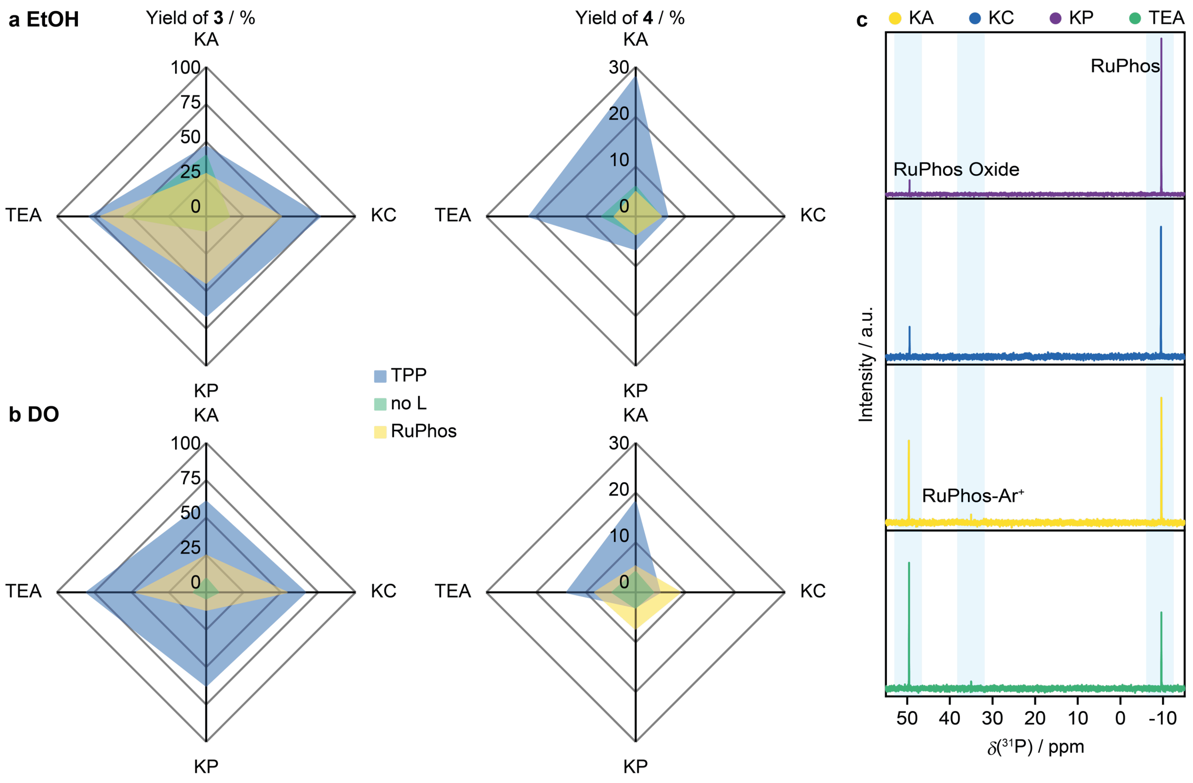

Figure S13. Effect of the absence of TPP or the presence of RuPhos instead of TPP on the yield of products **3** (left) and **4** (right) in **a** ethanol or **b** dioxane. **c** ^{31}P-NMR spectra of crude reaction mixtures with RuPhos instead of TPP.

References

(1) Faust Akl, D.; Poier, D.; D'Angelo, S. C.; Araújo, T. P.; Tulus, V.; Safonova, O. V.; Mitchell, S.; Marti, R.; Guillén-Gosálbez, G.; Pérez-Ramírez, J. Assessing the Environmental Benefit of Palladium-Based Single-Atom Heterogeneous Catalysts for Sonogashira Coupling. *Green Chem.* **2022**, *24* (18), 6879–6888. https://doi.org/10.1039/d2gc01853e.

(2) Büchele, S.; Zichittella, G.; Kanatakis, S.; Mitchell, S.; Pérez-Ramírez, J. Impact of Heteroatom Speciation on the Activity and Stability of Carbon-Based Catalysts for Propane Dehydrogenation. *ChemCatChem* **2021**, *13* (11), 2599–2608. https://doi.org/10.1002/cctc.202100208.

(3) Rivero-Crespo, M. A.; Toupalas, G.; Morandi, B. Preparation of Recyclable and Versatile Porous Poly(Aryl Thioether)s by Reversible Pd-Catalyzed C–S/C–S Metathesis. *J. Am. Chem. Soc.* **2021**, *143* (50), 21331–21339. https://doi.org/10.1021/jacs.1c09884.

(4) OriginPro, Version *2021*; OriginLab Corporation, Northampton, MA, USA.
